# UCK2 promotes intrahepatic cholangiocarcinoma progression and desensitizes cisplatin treatment by PI3K/AKT/mTOR/autophagic axis

**DOI:** 10.1038/s41420-024-02140-x

**Published:** 2024-08-23

**Authors:** Xiwen Wu, Da Chen, Muqi Li, Gehao Liang, Huizhen Ye

**Affiliations:** 1 Department of Clinical Nutrition, Sun Yat-sen University Cancer Center; State Key Laboratory of Oncology in South China; Guangdong Provincial Clinical Research Center for Cancer, Collaborative Innovation Center for Cancer Medicine, Guangzhou, PR China; 2 Department of Intensive Care Unit, Sun Yat-sen University Cancer Center; State Key Laboratory of Oncology in South China; Guangdong Provincial Clinical Research Center for Cancer, Collaborative Innovation Center for Cancer Medicine, Guangzhou, PR China; 3 Department of General Surgery, The First Affiliated Hospital of Nanchang University, Nanchang, Jiangxi PR China; 4 Department of Breast Surgery, Sun Yat-sen University Cancer Center; State Key Laboratory of Oncology in South China; Guangdong Provincial Clinical Research Center for Cancer, Collaborative Innovation Center for Cancer Medicine, Guangzhou, PR China; 5 Staff and Faculty Clinic, Sun Yat-sen University Cancer Center; State Key Laboratory of Oncology in South China; Guangdong Provincial Clinical Research Center for Cancer, Collaborative Innovation Center for Cancer Medicine, Guangzhou, PR China

**Keywords:** Cancer therapy, Oncogenes

## Abstract

Intrahepatic cholangiocarcinoma (iCCA) is a highly aggressive tumor with extremely poor prognosis due to the low resection rate, high recurrence rate and drug resistance. Uridine-cytidine kinase 2 (UCK2) is proved to promote progression and drug resistance of various carcinomas by regulating pyrimidine metabolism. However, the role of UCK2 in progression and drug resistance of iCCA was largely unclear. Gene expression matrices were obtained from public database and were verified by qRT-PCR using tumor sample from Sun Yat-sen University Cancer Center. Knockdown and overexpression of UCK2 were used to evaluate the effects of UCK2 on carcinogenesis and cisplatin response in iCCA. CCK8-kit assays and plate clone formation assays were performed to detect the effect of UCK2 on proliferative activity of tumor cells. Western blotting was performed to investigate protein level of UCK2 and the relevant biomarkers of PI3K/AKT/mTOR/autophagic axis. Cell migration and invasion were assessed by using wound-healing and transwell assays. UCK2 expression was detected elevated in iCCA tissues compared with adjacent normal tissues. Biologically, overexpression of UCK2 can promote proliferation of iCCA cells, and desensitizes iCCA to cisplatin in both in vivo and in vitro models. Mechanistically, UCK2 promote iCCA progression and cisplatin resistance through inhibition of autophagy by activating the PI3K/AKT/mTOR signaling pathway. Clinically, higher UCK2 expression in iCCA tumor was associated with aggressive tumor features, poorer survival and lower sensitivity of chemotherapy. UCK2 promotes iCCA progression and desensitizes cisplatin treatment by regulating PI3K/AKT/mTOR/autophagic axis. UCK2 exhibited potential as a biomarker in predicting prognosis and drug sensitivity of iCCA patients.

## Introduction

Intrahepatic cholangiocarcinoma (iCCA) is the second common primary liver cancer with an increasing morbidity in the last decade [1–4]. Unlike hepatocellular carcinoma (HCC), most iCCA developed in non-cirrhotic livers and no risk factors have been identified, making it hard to diagnose [2, 3, 5, 6]. Therefore, most iCCA patients are at an advanced stage at diagnosis, and lose the chance to receive curative resection. Beside curative resection, platinum-based chemotherapy is the main systemic treatment to eliminate micro-metastases and reduce the risk of recurrence [7, 8]. Despite standard treatment, a significant proportion of patients still suffer from recurrence and tumor-induced death, in part due to resistance of platinum-containing chemotherapy and lacking of therapeutic targets when facing drug resistance. Therefore, it is of great importance to identify novelty therapeutic targets and reveal underlying mechanism of drug resistance of iCCA.

Uridine-cytidine kinase (UCK), a rate-limiting enzyme in the rescue pathway of pyrimidine-nucleotide biosynthesis, are proved to promote progression and drug resistance of various carcinomas. There are two UCK genes (UCK1 and UCK2) in human being, and the catalytic functions efficacy of UCK2 is 15-20 times higher than that of UCK1, for which UCK2 show more power in biological function regulation [9]. UCK2 upregulation has been reported in several types of cancers including lung cancer [10, 11], breast cancer [12, 13], neuroblastoma [14, 15], colorectal cancer [16], pancreatic cancer [17], and HCC [18–21]. UCK2 expression was positively associated with poor overall survival in HCC [19–21], pancreatic cancer [22], and lung cancer [11]. Moreover, functional studies have confirmed that knockdown of UCK2 can inhibit cell proliferation and migration of HCC by Stat3 pathway and EGFR/AKT pathway [18, 19]. Totally, UCK2 may be involved in the malignant progression of various tumors in multiple pathways. However, the role of UCK2 in iCCA has not been fully investigated.

Here, we found that UCK2 expression was elevated in iCCA tissues, especially in chemo-resistant iCCA tissues, and high UCK2 expression was associated with aggressive tumor features and poor prognosis, indicating UCK2 show potential to be biomarker in predicting prognosis of iCCA patients. Biologically, we demonstrated that overexpression of UCK2 can activate the PI3K/AKT/mTOR signaling pathway in iCCA and desensitizes iCCA to cisplatin. Mechanistically, UCK2 transcription mediate iCCA progression through inhibition of autophagy by activating the PI3K/AKT/mTOR signaling pathway.

Autophagy is a cellular process that intracellular organelles and proteins are encapsulated into vesicles and fused with lysosomes to form autophagic lysosomes to degrade to meet the metabolic needs of cells themselves and renew some organelles [23, 24]. The activation or inhibition of autophagy plays an essential role in tumorigenesis and drug resistance [25, 26]. Interestingly, we found that UCK2 desensitizes iCCA to cisplatin by impairing autophagic level of iCCA cells.

We believe that UCK2 could be a potential novelty target to reverse cisplatin resistance of iCCA and the combined targeting of PI3K/AKT/mTOR signaling pathway and UCK2 has a potential to involve into the clinical use for iCCA patients.

## Results

### UCK2 was upregulated in iCCA tissues and was associated with the poor prognosis of iCCA patients

**Fig. 1:**
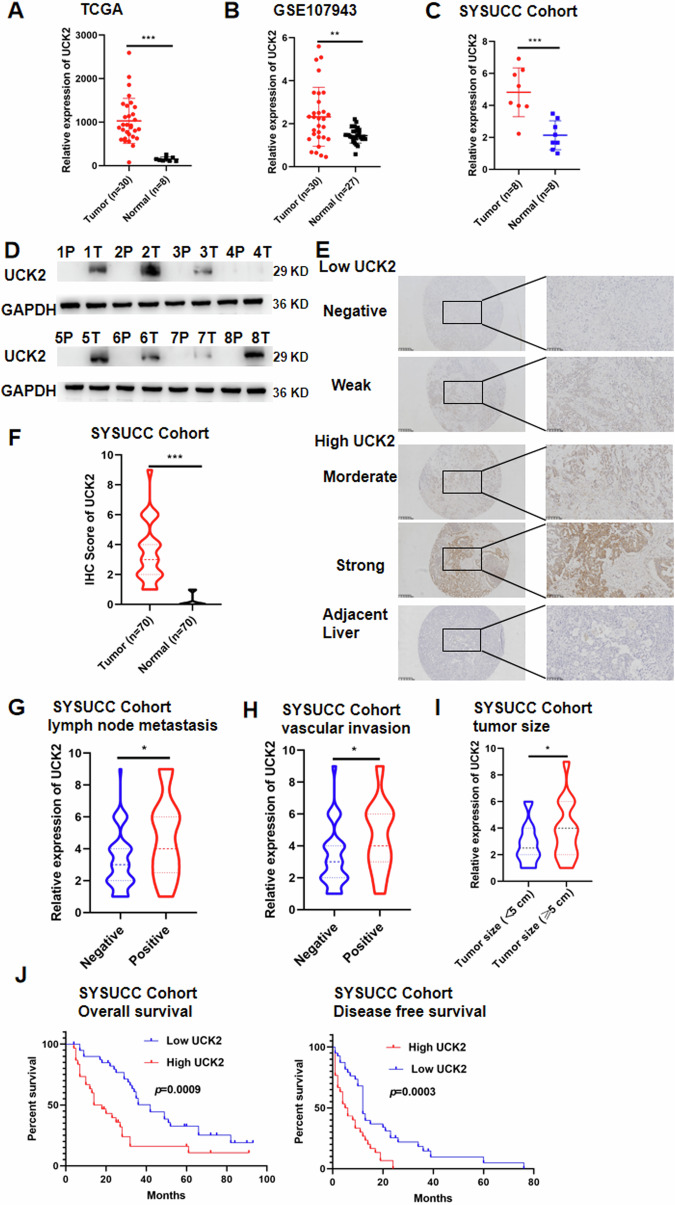
UCK2 is upregulated in iCCA and is associated with poor prognosis. **A** CK2 expression of iCCA tumor (*n* = 36) and normal tissue (*n* = 8) in TCGA database. **B** UCK2 expression of iCCA tumor (*n* = 30) and normal tissue (*n* = 27) in public dataset GSE107943. **C** UCK2 expression of iCCA tumor (*n* = 8) and normal tissue (*n* = 8) were measured by real-time quantitative reverse transcription polymerase chain reaction (qRT-PCR) from SYSUCC Cohort. **D** UCK2 expression of iCCA tumor (*n* = 8) and normal tissue (n = 8) were measured by Western blotting from SYSUCC Cohort. **E** Representative immunohistochemistry (IHC) staining images of iCCA tumors expressing low or high levels of UCK2. **F** IHC scores of UCK2 in iCCA tumors and adjacent liver of 70 iCCA patients. **G** Correlation analysis of UCK2 expression with lymph node metastasis. **H** Correlation analysis of UCK2 expression with vascular invasion. **I** Correlation analysis of UCK2 expression with tumor size (≤5 cm vs. >5 cm). **J** Kaplan–Meier survival curve of overall survival (OS) and disease-free survival (DFS) in 70 iCCA patients, stratified by UCK2 IHC score (UCK2 low expression, *n* = 40 vs. UCK2 high expression, *n* = 30). The *P* value was calculated using the log-rank test. *P < 0.05, **P < 0.01, ***P < 0.001, according to Student’s *t*-test.

We found that UCK2 was upregulated in iCCA tissues compared with the adjacent normal tissues in TCGA (Fig. 1A), GEO dataset GSE107943 (Fig. 1B) and in Sun Yat-sen University Cancer Center Cohort (SYSUCC Cohort) (Fig. 1C). Furthermore, UCK2 expression levels was higher in tumor tissues than that in normal tissues specimens by western blotting in SYSUCC Cohort (Fig. 1D). We then measured the expression of UCK2 in 70 iCCA tumor tissues and matched adjacent liver tissues by immunohistochemistry (IHC) staining (Fig. 1E) and found that UCK2 expression were higher in iCCA tumor tissues than matched adjacent liver tissues (*P* < 0.001) (Fig. 1F). Then, we explored the relationship between UCK2 expression and clinical-pathological characteristics or patients survival in 70 iCCA patients. UCK2 expression was positively correlated with lymph node metastasis (*P* < 0.05) (Fig. 1G), vascular invasion (*P* < 0.05) (Fig. 1H) and large tumor size (*P* < 0.05) (Fig. 1I). Survival analysis showed that high UCK2 expression was correlated with poor overall survival (*P* < 0.001) (Fig. 1J) and disease-free survival (*P* < 0.001) (Fig. 1J).

### Downregulation of UCK2 significantly suppresses iCCA progression

**Fig. 2:**
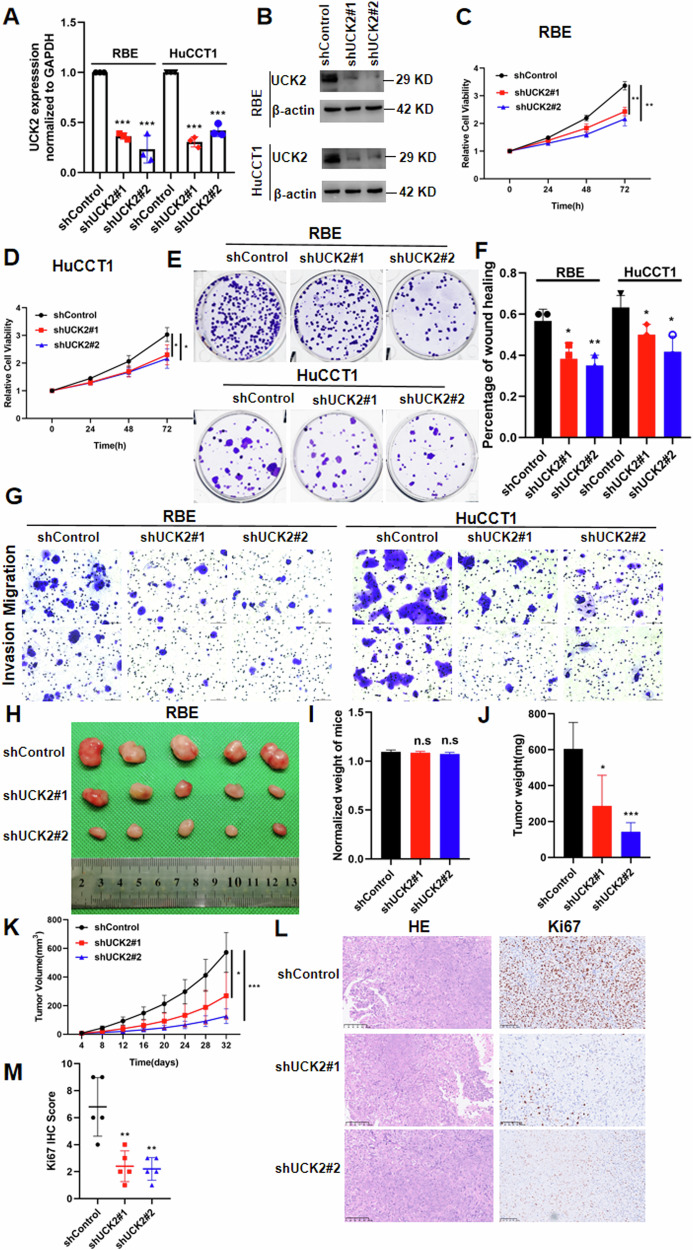
UCK2 depletion suppresses iCCA progression. **A** The mRNA level of UCK2 after UCK2 knockdown was confirmed by qRT-PCR. **B** The protein level of UCK2 after UCK2 knockdown was confirmed by western blotting. **C**, **D** Cell growth curve of RBE and HuCCT1 cells transfected with UCK2 shRNA or control. **E** Colony-forming assays after UCK2 knockdown in HuCCT1 and RBE cells. **F** Wound-healing assays after UCK2 silencing in HuCCT1 and RBE cells. **G** Cell migration ability and cell invasion ability after shUCK2-transfection in RBE and HuCCT1 cell. **H** Xenograft tumors in each group were shown. The mice were sacrificed 32 days post-injection. **I** Body weight of shUCK2 and Control groups was measured. **J** Tumor weight of shUCK2 and Control groups was measured. **K** Tumor growth curves after the injection of shUCK2 and Control RBE cells and Tumor volume was calculated every 4 days. **L**, **M** IHC staining of Ki67 in tumors of RBE shUCK2 and Control group. *P < 0.05, **P < 0.01, ***P < 0.001, according to Student’s *t*-test.

**Fig. 3:**
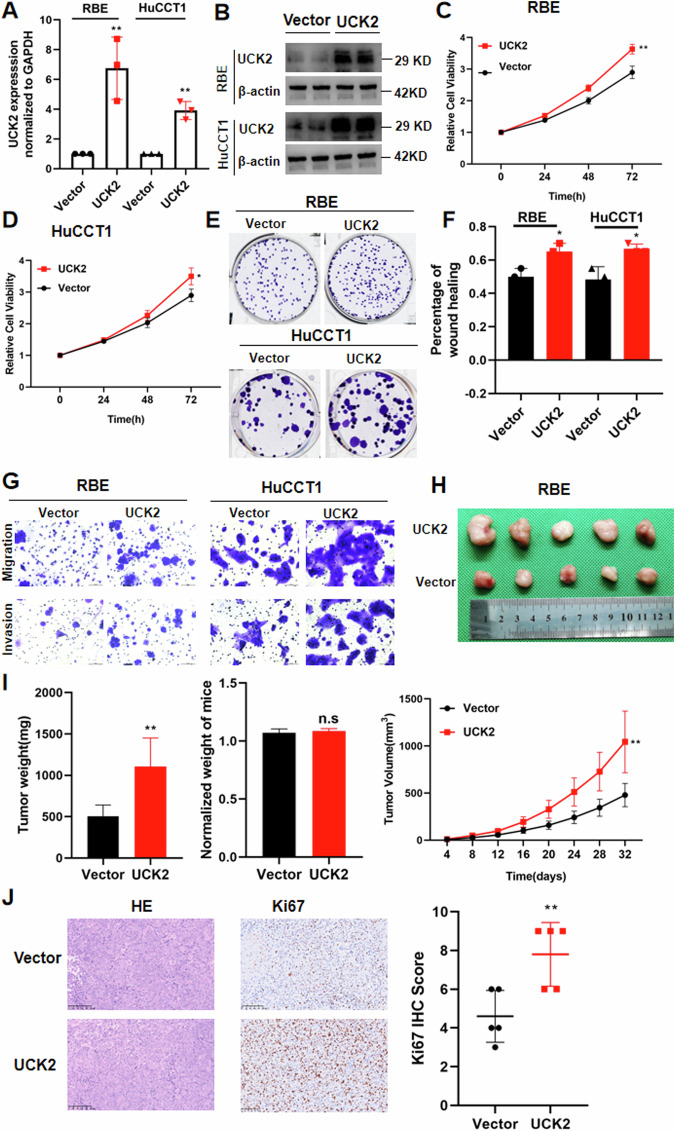
UCK2 overexpression promotes iCCA progression. **A** The mRNA level of UCK2 after UCK2 overexpression was confirmed by qRT-PCR. **B** The protein level of UCK2 after UCK2 overexpression was confirmed by western blotting. **C**, **D** Cell growth curve of RBE and HuCCT1 cells with UCK2 overexpression or control. **E** Colony-forming assays after UCK2 overexpression in HuCCT1 and RBE cells. **F** Wound-healing assays after UCK2 overexpression in HuCCT1 and RBE cells. **G** Cell migration ability and cell invasion ability after UCK2 overexpression in RBE and HuCCT1 cell. **H** Xenograft tumors in each group were shown. The mice were sacrificed 32 days post-injection. **I** Body weight and tumor weight of UCK2 overexpression and control groups was measured. Tumor growth curves after the injection of UCK2 overexpression and control RBE cells was calculated every 4 days. **J** IHC staining of Ki67 in tumors of UCK2 overexpression and Control group. *P < 0.05, **P < 0.01, ***P < 0.001, according to Student’s *t*-test.

To explore the function of UCK2 in iCCA progression, we then constructed stable UCK2-knockdown RBE and HuCCT1 cell lines using lentivirus carrying shRNA. The knockdown efficiency of UCK2 was verified by qPCR (Fig. 2A) and western blotting (Fig. 2B). Knockdown of UCK2 significantly inhibited iCCA cells proliferation (Fig. 2C/D) and colony formation (Fig. 2E). The cell scratching assay showed that UCK2 knockdown reduced RBE and HuCCT1 migration ability (Fig. 2F). Transwell migration and invasion assays showed that UCK2 knockdown significantly reduced the ability of migration and invasion of iCCA cells (Fig. 2H). UCK2-knockdown RBE cells and control cells were further subcutaneously injected into nude mice to verify the effect of UCK2 on iCCA growth in vivo. UCK2-knockdown tumors grew slowly than the tumors of control group (Fig. 2H). And the body weight of the three groups showed no significant difference (Fig. 3I). The tumor weight (Fig. 2J) and tumor volume (Fig. 2K) was markedly lower in UCK2-knockdown group than the control group. In addition, Immunohistochemistry (IHC) results showed Ki67 score in UCK2-knockdown group was significantly decreased compared with that in the control group (Fig. 2L/M).

### UCK2 overexpression promotes iCCA progression

To further validate the function of UCK2 in iCCA progression, we then constructed stable UCK2 overexpression RBE and HuCCT1 cell lines using lentivirus carrying UCK2. The overexpression efficiency of UCK2 was verified by qPCR (Fig. 3A) and western blotting (Fig. 3B). UCK2 overexpression significantly promotes iCCA cells proliferation (Fig. 3C/D) and colony formation (Fig. 3E). The cell scratching assay showed that UCK2 overexpression promotes RBE and HuCCT1 migration ability (Fig. 2F). Migration and invasion assays showed that UCK2 overexpression promotes the ability of migration and invasion of iCCA cells (Fig. 2H). UCK2 overexpression RBE cells and control cells were further subcutaneously injected into nude mice to verify the impact of UCK2 on iCCA growth in vivo. Tumors from UCK2 overexpression group grew bigger than that of control group (Fig. 3H/I). And the body weight of the two groups showed no significant difference (Fig. 3I). The tumor weight and tumor volume (Fig. 3I) was markedly higher in UCK2-overexpression group than the control group. In addition, IHC staining results showed the expression of Ki67 in UCK2 overexpression group was significantly higher than that in the control group (Fig. 3J).

### UCK2 facilitates iCCA progression by activating the PI3K/AKT/mTOR signaling pathway

**Fig. 4:**
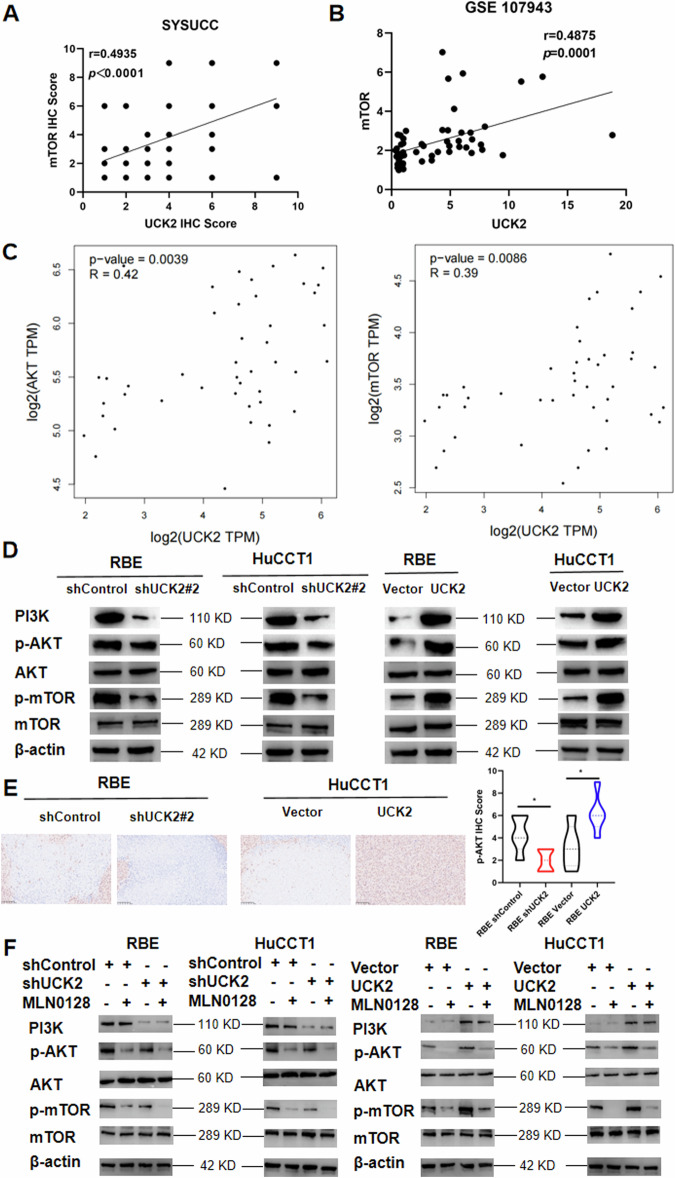
UCK2 facilitates iCCA progression by activating the PI3K/AKT/mTOR signaling pathway. **A** UCK2 positively correlates with mTOR analyzed by SUSUCC Cohort. **B** UCK2 positively correlates with mTOR analyzed by GSE107943. **C** UCK2 positively correlates with AKT and mTOR analyzed by GEPIA2. **D** The PI3K, phosphorylated AKT (p-AKT), phosphorylated-mTOR (p-mTOR) and AKT and mTOR protein levels after UCK2 knockdown or overexpression. **E** IHC staining of p-AKT in tumors of each group. **F** The PI3K, AKT, mTOR, p-AKT and p-mTOR protein levels after UCK2 knockdown or overexpression plus MLN0128. *P < 0.05, **P < 0.01, ***P < 0.001, according to Student’s t test.

mTOR expression was positively associated with UCK2 expression in SYSUCC cohort according to the IHC score (Fig. 4A). mTOR mRNA expression was positively associated with UCK2 mRNA expression in GEO database GSE107943 (Fig. 4B). Furthermore, AKT and mTOR was positively correlated with UCK2 analyzed by GEPIA2 according to the TCGA dataset (Fig. 4C). We then use western blotting to validate the protein level of the key markers of the PI3K/AKT/mTOR pathway. The expression of phosphorylated protein of the AKT and mTOR in shUCK2 cells were significantly decreased than the Control cells, while the expression of phosphorylated protein of the AKT and mTOR in UCK2 overexpression cells were significantly higher than that in Control-Vector cells (Fig. 4D). This finding was further confirmed by p-AKT IHC staining using the mice xenograft tumors (Fig. 4E). Results shown that after MLN0128 (mTOR1/2 inhibitor) treatment, the phosphorylated protein of the AKT and mTOR was significantly decreased, while the expression of the PI3K, AKT and mTOR was with no significantly difference (Fig. 4F). UCK2 knockdown plus MLN0128 treatment revealed the least protein level of phosphorylated AKT and mTOR (Fig. 4F).

Results shown that after AKT inhibitor MK-2206 treatment, the phosphorylated protein of the AKT and mTOR was significantly decreased, while the expression of the PI3K, AKT and mTOR was with no significantly difference (Fig. S1A). UCK2 knockdown plus MK-2206 treatment revealed the least protein level of phosphorylated AKT and mTOR (Fig. S1A). What’s more, results shown that after PI3K inhibitor GDC-0941 treatment, PI3K, the phosphorylated protein of the AKT and mTOR was significantly decreased, while the expression of AKT and mTOR was with no significantly difference (Fig. S1B). UCK2 knockdown plus GDC-0941 treatment revealed the least protein level of phosphorylated AKT and mTOR (Fig. S1B).

### UCK2 overexpression mediate iCCA progression through inhibition of autophagy by activating the PI3K/AKT/mTOR signaling pathway

**Fig. 5:**
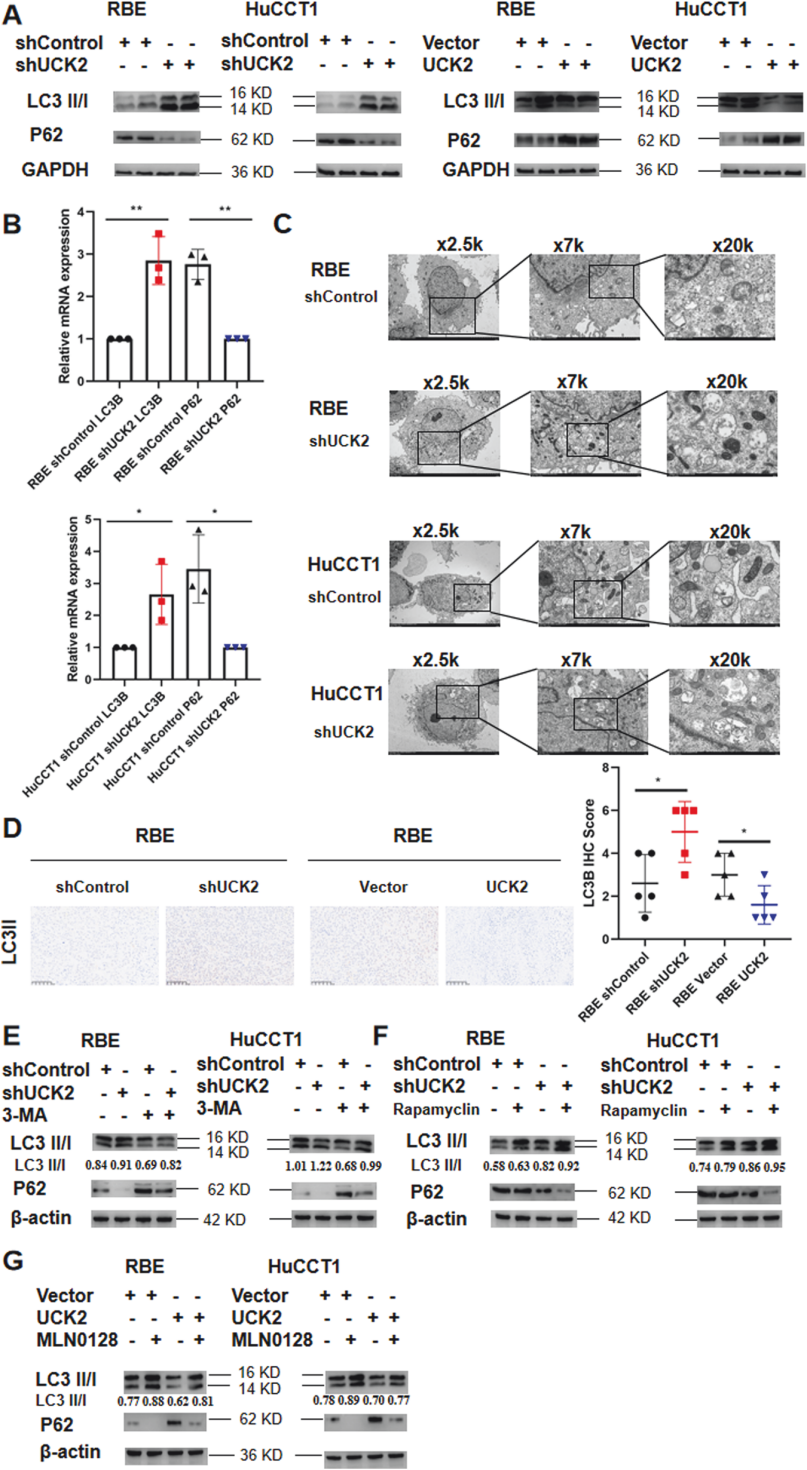
UCK2 overexpression mediate iCCA progression through inhibition of autophagy by activating the PI3K/AKT/mTOR signaling pathway and dual inhibition of PRIM1 and mTOR enhance Autophagy on iCCA cells. **A** Relative protein levels of LC3II/I, p62 and beclin-1 in RBE and HuCCT1 cells after silencing UCK2 or overexpressing UCK2. **B** Relative mRNA expression of LC3B and p62 in RBE and HuCCT1 cells after silencing UCK2. **C** Transmission electron microscopy (TEM) pictures for detecting the autophagosomes RBE and HuCCT1 cells after silencing UCK2 (×2500/3000, 7000/8000 and 20000 magnification). **D** IHC staining of LC3II in tumors of each group. **E** Relative protein levels of LC3II/I and p62 in RBE and HuCCT1 cells after silencing UCK2 puls 3-MA. **F** Relative protein levels of LC3II/I and p62 in RBE and HuCCT1 cells after silencing UCK2 puls Rapamyclin. **G** Relative protein levels of LC3II/I and p62 in RBE and HuCCT1 cells after UCK2 overexpression puls MLN0128. *P < 0.05, **P < 0.01, ***P < 0.001, according to Student’s t test.

We then detected the effect of UCK2 expression on autophagy to explain the underlying mechanisms that UCK2-mediated progression of iCCA since previous studies have indicated that PI3K/AKT/mTOR signaling pathway is highly associated with autophagy. First, To confirm the effect of UCK2 on autophagy, we examine the expression of LC3II/I, P62 and Beclin1 in iCCA cells by western blotting. UCK2 knockdown increased LC3 II/I and beclin1 expression and decreased p62 expression, while the overexpression of UCK2 decreased LC3II/I and Beclin1 expression and increased p62 expression (Fig. 5A). The results were further confirmed by qPCR that LC3B (LC3II) mRNA were higher expressed in UCK2 knockdown cells, while the P62 mRNA were higher expressed in UCK2 shControl cells (Fig. 5B). Second, transmission electron microscopy (TEM) results showed that there were more autophagosomes in UCK2 knockdown cells than UCK2 shControl cells (Fig. 5C). The result was further validated by LC3B IHC staining with mice xenograft tumors (Fig. 5D).

### Dual inhibition of UCK2 and mTOR enhance autophagy on iCCA cells

We then investigated the effect of combined targeting of UCK2 and mTOR on iCCA. Results showed that UCK2 knockdown-mediated autophagy effect were reversed when shUCK2 iCCA cells were co-treated with the autophagy inhibitor 3-MA (Fig. 5E). Co-treatment with 3-MA significantly decreased UCK2 inhibition-dependent LC3 II/I and beclin1 protein expression (Fig. 5E). We found that rapamycin (mTOR inhibitor) alone or UCK2 knockdown alone increased LC3 II/I and beclin1 protein expression while decreased p62 protein expression compared to that of the control group (Fig. 5E). Dual inhibition of UCK2 and mTOR showed and enhanced effect of increasing LC3 II/I expression while decreasing p62 protein expression (Fig. 5F). We also found that MLN0128 (mTOR inhibitor) alone increased LC3 II/I and beclin1 protein expression while decreased p62 protein expression compared to that of the control group (Fig. 5G). UCK2 overexpression plus MLN0128 enhanced effect of increasing LC3 II/I expression while decreasing p62 protein expression compared with UCK2 overexpression alone (Fig. 5G).

### Overexpression of UCK2 desensitizes iCCA cells to cisplatin

**Fig. 6:**
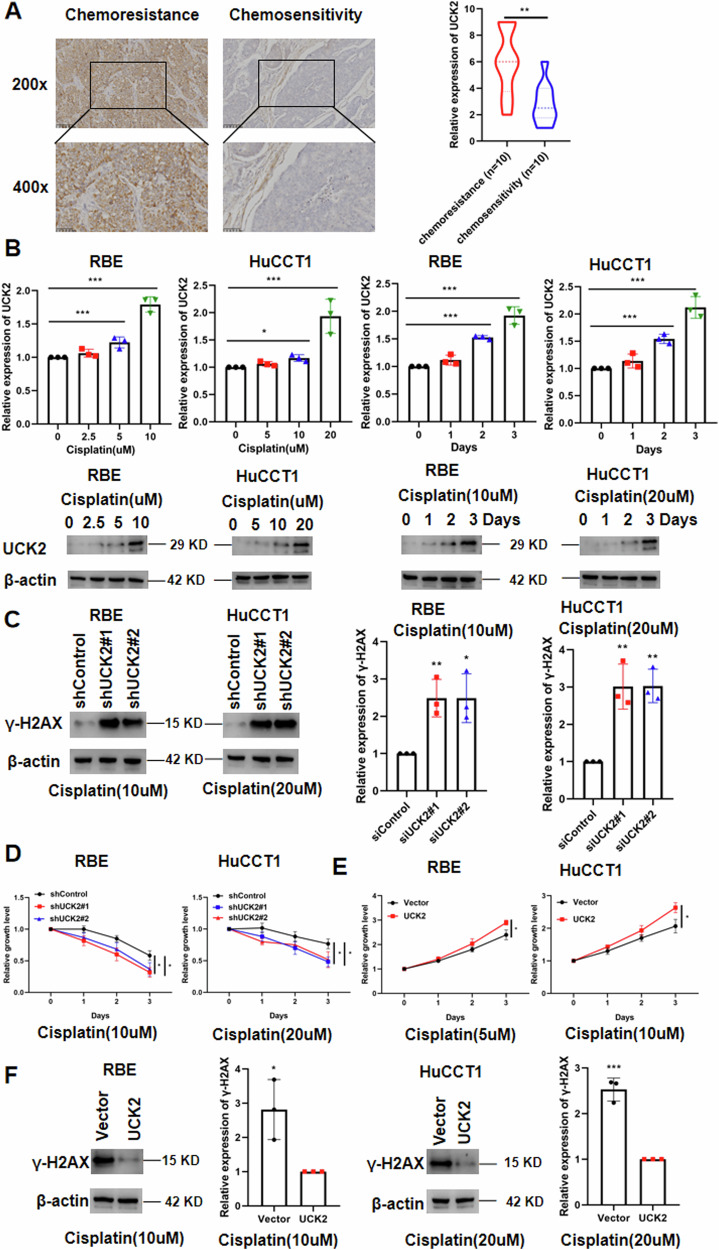
Overexpression of UCK2 desensitizes iCCA cells to cisplatin. **A** Representative immunohistochemistry (IHC) staining images and IHC scores for UCK2 in 10 chemo-resistant and 10 chemo-sensitive iCCA tissues. **B** Changes of UCK2 expression in both a dose and time manner in RBE and HuCCT1 cells after cisplatin treatment. **C** Protein expression and mRNA expression of γ-H2AX in shControl or shUCK2-transfected HuCCT1 and RBE cells after treatment with cisplatin (20 or 10 μM, respectively). **D** Cell viability of shControl or shUCK2-transfected HuCCT1 and RBE cells after treatment with cisplatin (10 or 5 μM, respectively). **E** Cell viability of Control or UCK2 overexpression transfected HuCCT1 and RBE cells after treatment with cisplatin (20 or 10 μM, respectively). **F** Protein expression and mRNA expression of γ-H2AX in Control or UCK2 overexpression transfected HuCCT1 and RBE cells after treatment with cisplatin (10 or 5 μM, respectively). *P < 0.05, **P < 0.01, ***P < 0.001, according to Student’s *t*-test.

**Fig. 7:**
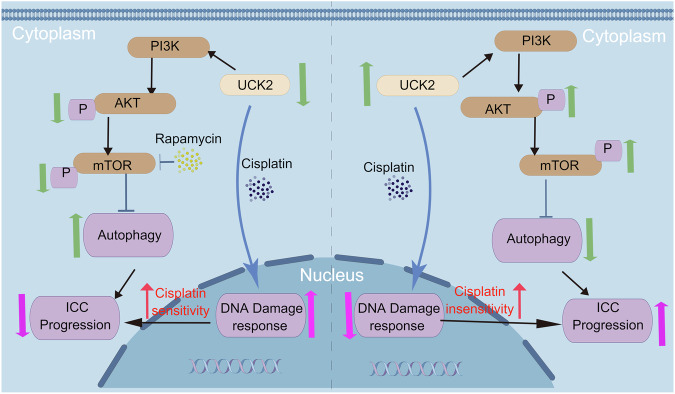
The schematic diagram for function and mechanism of UCK2 on iCCA. (Right) In iCCA cells, UCK2 overexpression mediated ICC progression through inhibition of autophagy by activating the PI3K/AKT/mTOR signaling pathway and desensitized iCCA to cisplatin treatment via decreasing DNA damage response. (Left) dual inhibition of UCK2 and mTOR suppressed the function of PI3K/AKT/mTOR signaling pathway, then enhance autophagy on iCCA cells resulting in tumor growth inhibition. The schematic diagram was draw by FigDraw (export ID:TPATUf6b66). *P < 0.05, **P < 0.01, ***P < 0.001, according to Student’s t test.

To test the function of UCK2 on the cisplatin treatment of iCCA, the expression of UCK2 in 10 chemo-resistant and 10 chemo-sensitive iCCA tissues was detected by IHC staining. We found a higher expression of UCK2 in chemo-resistant patients with iCCA than in sensitive patients (Fig. 6A). We then assessed changes of UCK2 expression after cisplatin treatment. We found that cisplatin treatment induced the upregulation of UCK2 in both a dose-dependent and time-dependent manner in RBE and HuCCT1 cells (Fig. 6B). Downregulation of UCK2 increased the DNA damage to cisplatin treatment in HuCCT1 and RBE cells (Fig. 6C). As expected, downregulation of UCK2 can increase the sensitivity of HuCCT1 and RBE cells to cisplatin treatment (Fig. 6D). We then tested whether overexpression of UCK2 had the opposite effect to cisplatin treatment in iCCA cells. As expected, overexpression of UCK2 desensitized HuCCT1 and RBE cells to cisplatin treatment (Fig. 6E). Overexpression of YTHDF2 decreased the DNA damage to cisplatin treatment in HuCCT1 and RBE cells (Fig. 6F). These findings suggest that UCK2 contributes to desensitization against cisplatin in iCCA (Fig. 7).

## Discussion

iCCA is a highly aggressive tumor with extremely poor prognosis due to the low resection rate, high recurrence rate and drug resistance [1, 27]. Cisplatin is the current baseline chemotherapy drug for advanced iCCA [8]. However, iCCA resistance to cisplatin remains one of the hard challenge in the comprehensive treatment of iCCA. Pyrimidine metabolism is important for tumor progression and UCK is a rate-limiting enzyme in the rescue pathway of pyrimidine-nucleotide biosynthesis. UCK2, the core component of UCK, has a higher catalytic efficiency than UCK1, indicating that UCK2 is a potential chemotherapeutic drug target [9]. However, the molecular mechanism involving UCK2 associated with cancer progression and chemoresistance in iCCA remains unclear.

Here, we found that UCK2 expression was elevated in iCCA tissues, especially in chemo-resistant iCCA tissues, and high UCK2 expression was associated with aggressive tumor features and poor prognosis, indicating UCK2 show potential to be biomarker in predicting prognosis of iCCA patients. Biologically, we demonstrated that overexpression of UCK2 can activate the PI3K/AKT/mTOR signaling pathway in iCCA and desensitizes iCCA to cisplatin. Mechanistically, UCK2 transcription mediate iCCA progression through inhibition of autophagy by activating the PI3K/AKT/mTOR signaling pathway. Dual inhibition of UCK2 and mTOR enhance Autophagy on iCCA cells. Therefore, inhibition of UCK2 would be an attractive strategy to sensitize iCCA cells to chemotherapy.

UCK2, an important rate-limiting enzyme in the rescue pathway of pyrimidine-nucleotide biosynthesis, exhibits a series of biological function of promoting cancer proliferation, invasion and metastasis. Zhou et al. found that UCK2 promotes HCC cell migration, invasion and metastasis via the Stat3 signaling pathway and might be a novel potential target in HCC therapy [18]. Wu et al. reported that downregulation of UCK2 induces cell cycle arrest and activates the TNFα/NF-κB signaling pathway-related senescence-associated secretory phenotype to modify the tumor microenvironment and could improve the response to immunotherapy in patients with HCC [28]. UCK2 overexpression promotes cancer cell proliferation and metastasis by activating the Wnt/β-catenin [29]. UCK2 can non-metabolically activating EGFR-AKT signaling pathway to promote HCC progression and may provide novel UCK2-based therapeutic strategies for cancer treatment [19]. Further, UCK2 was upregulated and played as an oncogenic role in HCC, pancreatic cancer, lung cancer, testicular germ cell tumors and endometrial cancer [30]. However, the role of UCK2 in iCCA carcinogenesis has not yet been investigated. Our study first implied that UCK2 play a critical role in iCCA progression via the activation PI3K/AKT/mTOR signaling pathway, thereby inhibiting the autophagy of iCCA cells.

Cisplatin is one of the most widely used chemotherapy drug in various tumors and combination therapy based on cisplatin has become the first-line treatment for advanced or metastatic iCCA patients. Previous studies have reported a close relation between tumor UCK2 expression and the objective response to those cytotoxic ribonucleoside analogs. UCK2 were closely associated with cellular sensitivity to TAS-106 and it may contribute to the tumor-selective cytotoxicity of TAS-106 [17]. Fluorocyclopentenylcytosine (RX-3117) is activated by UCK2 and accumulation of RX-3117 nucleotides correlated with UCK2 expression [31]. What’s more, UCK2 could serve as a prospective biomarker of potential response to RX-3117 treatment in pancreatic cancer patients [22]. UCK2 upregulated in cancer is partly due to demethylation, which contributes to resistance of cancer to 5-azacytidine treatment [32]. The role of UCK2 in iCCA cisplatin treatment has not been yet investigated. The results of present study revealed that UCK2 is upregulated in chemo-resistant iCCA tissues. Mechanistically, UCK2 transcription mediate iCCA progression through inhibition of autophagy by activating the PI3K/AKT/mTOR signaling pathway and desensitizes iCCA cells to cisplatin treatment.

mTOR, a downstream mediator of PI3K/AKT pathway, is a vital regulation pathway of cellular autophagy. The activation or inhibition of autophagy plays an essential role in tumorigenesis [25, 26]. Previous study confirmed that UCK2 is essential for maintaining the stability of mTOR and downregulation of UCK2 can specifically inhibit mTOR signaling pathway-related metabolic reprogramming of cancer cells [28]. In our study, we demonstrated that overexpression of UCK2 can activate the PI3K/AKT/mTOR signaling pathway in iCCA and desensitizes iCCA to cisplatin. Here, we observe that UCK2 knockdown increased LC3 II/I and beclin1 expression and decreased p62 expression, which suggests that UCK2 knockdown facilitates autophagy. Dual inhibition of UCK2 and mTOR showed and enhanced effect of increasing LC3 II/I and beclin1 protein expression while decreasing p62 protein expression. Cancer treatment strategies that alter cancer metabolism may improve cancer therapy.

Several limitations of the study were as follows. First, most of the findings were derived from western blotting results. Second, the chemo-resistant and chemo-sensitive iCCA sample size were relatively small. Third, the cisplatin resistance experiment was only limited to cell experiment and further animal experiments are needed to verify the results.

Therefore, based on these results, we proposed that UCK2 can promote ICC tumorigenesis and desensitize ICC to cisplatin treatment.

## Material and methods

### Patients and follow-up

A total of 70 iCCA patients who had signed the informed consent from the Sun Yat-Sen University Cancer Center were enrolled and the study was approved by the Institutional Review Board and the Research Ethics Committee of Sun Yat-sen University Cancer Center. All enrolled patients have complete clinical-pathological characteristics and follow-up data which were used for subsequent survival analysis. The paraffin-embedded specimens of iCCA patients were used for UCK2 Immunohistochemistry experiments. Another 8 fresh iCCA tumor tissues and matched adjacent normal bile duct tissues for subsequent qRT-PCR of UCK2 were snap-frozen in liquid nitrogen within 30 min after surgical resection in our center.

### Public dataset analysis

We compared the mRNA expression of UCK2 between the two groups according to the GEPIA2 database which consisted of 36 iCCA cancer and 9 adjacent non-cancerous tissues samples. The GSE107943 were downloaded and the mRNA expression of UCK2 were compared between two groups.

### Immunohistochemistry (IHC)

The results of IHC staining assay were based on the intensity of staining (negative, 0; weak, 1; medium, 2; strong, 3) and the percentage of positive signal (negative, 0; 1–30%, 1; 30–60%, 2; above 60%, 3). Multiplying the two scores to get the immunoreactivity scores (IRSs) and high expression was defined as having IRS more than 3. The dilution ratio of anti-UCK2 antibody, anti-p-AKT, and anti-Ki67 were 1:200, 1:100 and 1:3000, respectively. Rabbit polyclonal anti-human UCK2 antibodies (1:100, Abcam, #ab241281, USA) were used for IHC according to the instructions.

### Cell lines and lentiviral transfection

Two human iCCA cells RBE and HuCCT1 were obtained from Guangzhou Cellcook Biotech Co., Ltd (Guangzhou, China) and were cultured under the condition of 37 °C and with 5% CO2 using RPMI-1640 medium (Gibco, USA) containing 10% fetal bovine serum (FBS) (Gibco, USA).

The UCK2 overexpression lentiviral vectors and the empty lentiviral vectors were transfected into iCCA cells and designated as RBE/HuCCT1-UCK2 cells and RBE/HuCCT1-Vector cells, while the short hairpin UCK2 lentiviral vectors and the empty lentiviral vectors were also transfected into iCCA cells designated as RBE/HuCCT1-shUCK2 cells and HuCCT1/RBE-Control cells (Shanghai Genechem Co., Ltd., China, customer order number: GOSL0262169).

### Western blotting and qRT-PCR

The UCK2 expression between iCCA tumor tissues and matched adjacent normal bile duct tissues were extracted and compared by western blotting and qRT-PCR. The anti-UCK2 antibody (#ab241281), anti-Ki67 (#ab156956) and anti-LC3B antibody (#ab192890) were purchased from Abcam. The GAPDH antibody (#2118), anti-rabbit IgG, HRP-linked antibody (#7071), anti-AKT (#4691), anti-phospho-AKT (#4060), anti-mTOR (#2983), anti-PI3K (#4249), anti-P62 (#39749), anti-Beclin1 (#3945) and anti-phospho-mTOR (#2971) were purchased from Cell Signaling Technology (Danvers, MA, USA). Total RNA was isolated from indicated cells using TRIzol reagent (Invitrogen, USA). Reverse transcription and quantitative Real-Time PCR (qRT-PCR) were performed using HiScript II Q RT SuperMix for qPCR (R223-01, Vazyme, China) and SYBR qPCR Master Mix (Q711-02, Vazyme, China), respectively. Relative expression was calculated by the 2^-ΔΔCt^ formula after being normalized to GAPDH expression.

### Cell viability, clone formation assay, wound-healing, and transwell migration experiments

Cell viability was tested using Cell Counting Kit-8 (CCK-8, DOJINDO, Japan) according to the manufacturer’s instructions. For clone formation assay, 1000 iCCA cells with or without UCK2 overexpression or knockdown were seeded into a 6-well plate and crystal violet staining was used for counting the cell colonies after culturing for 14 days. For the wound-healing experiment, A straight scratch was made by using a pipette tip and the cells were cultured for 48 h. For the transwell migration and invasion assay (with Matrigel, BD Biosciences, San Jose, CA, USA), 2 × 10^4^ iCCA cells were suspended in serum-free medium in the upper compartments and incubated for 48 h. And then the translocated cells were fixed by methanol and stained by crystal violet.

### Animal experiments

All animal studies were following the Guide for the Care and Use of Laboratory Animals (NIH publication nos. 80-23, revised 1996) and were approved by the Institutional Animal Care and Use Committee of Sun Yat-sen University Cancer Center. 5 × 10^6^ RBE cells with or without UCK2 knockdown or overexpression were resuspended in 100 ul PBS with Matrigel (1:1) and subcutaneously implanted into BALB/c nude female mice (4 weeks old, GemPharmatech Co., Ltd, Guangdong, the mice were randomized into 5 groups, 5 mice per group). The tumor size was measured every 4 days and the volume was calculated using the formula: *v* = length × width^2^/2. After 32 days of implantation, the mice were executed and xenograft tumors were removed, photographed and weighted.

### Statistical analysis

For the measurement data were as mean ± standard deviation, Student *t*-test was used for inter group comparison. Log-rank test and Kaplan–Meier analysis were used for survival analysis. Spearman’s correlation was used to assess the correlations between two groups. GraphPad Prism 9.0 software and SPSS v20.0 software for Windows (IBM, Chicago, IL, USA) were used for statistical analysis in this study, and *p* < 0.05 (two-tailed) was considered to have significant difference.

### Supplementary information

figure S1

Supplemental Material Revised

## Data Availability

The raw sequencing data analyzed in the current study is available from the corresponding author on reasonable request and full length western blots were uploaded with supplemental material file.
