# Supplementary figures and images for: UCK2 promotes intrahepatic cholangiocarcinoma progression and desensitizes cisplatin treatment by PI3K/AKT/mTOR/autophagic axis
Source: Cell Death Discov. 2024 Aug 23;10:375. doi: 10.1038/s41420-024-02140-x (PMC11344076; doi:10.1038/s41420-024-02140-x)

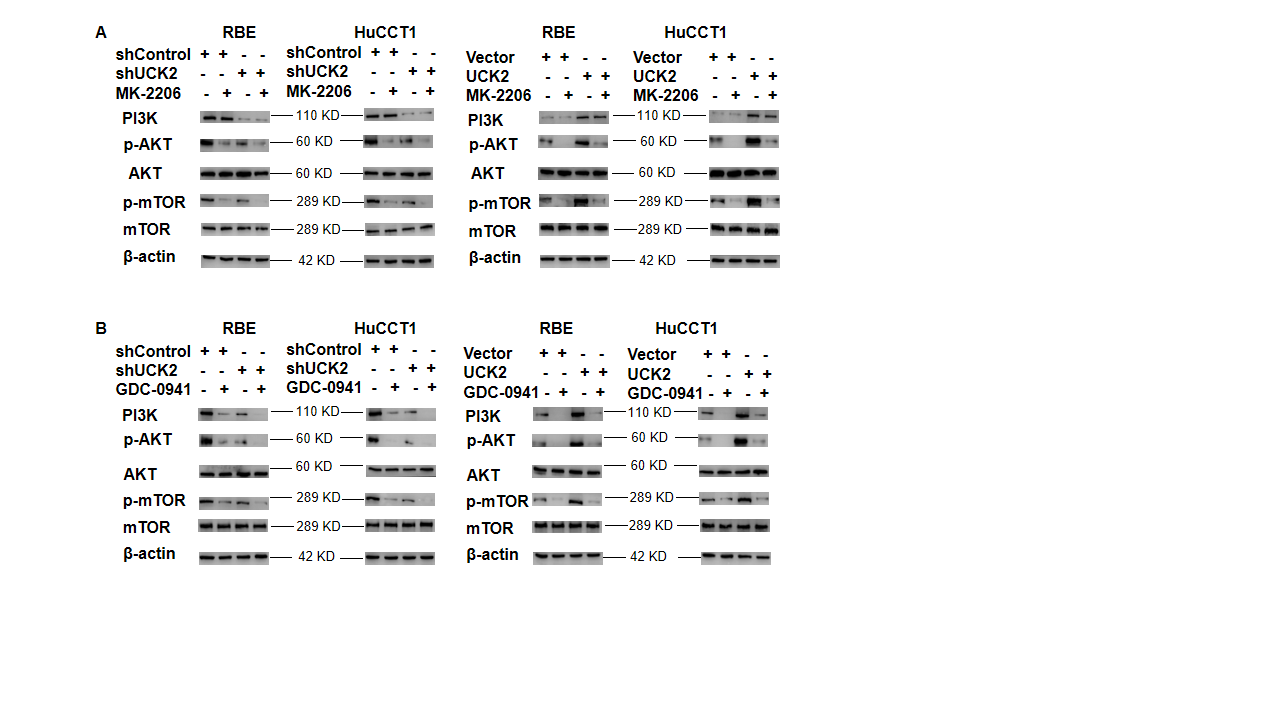

Supplement: Supplementary file 1 — figure S1 [file 41420_2024_2140_MOESM1_ESM.tif]
