# Supplementary material for: UCK2 promotes intrahepatic cholangiocarcinoma progression and desensitizes cisplatin treatment by PI3K/AKT/mTOR/autophagic axis
Source: Cell Death Discov. 2024 Aug 23;10:375. doi: 10.1038/s41420-024-02140-x (PMC11344076; doi:10.1038/s41420-024-02140-x)

Original Data

Supplementary legends

Figure S1. UCK2 facilitates iCCA progression by activating the PI3K/AKT/mTOR signaling pathway.

(A) The PI3K, AKT, mTOR, p-AKT and p-mTOR protein levels after UCK2 knockdown or overexpression plus AKT inhibitor MK-2206.

(B) The PI3K, AKT, mTOR, p-AKT and p-mTOR protein levels after UCK2 knockdown or overexpression plus PI3K inhibitor GDC-0941.

Original Data

Supplemental Material for western blots

Figure 1D

UCK2

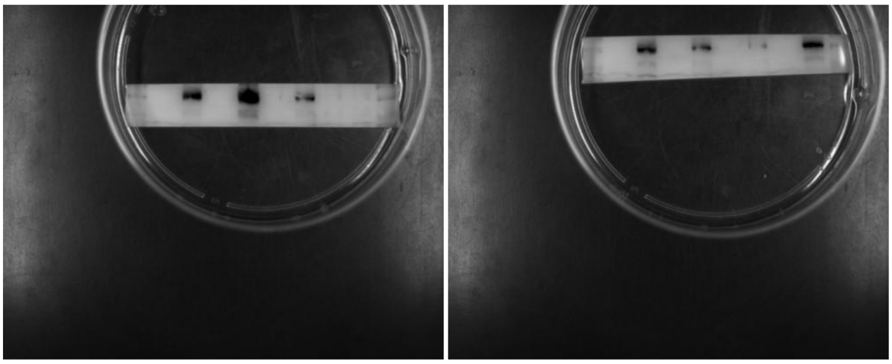

GAPDH

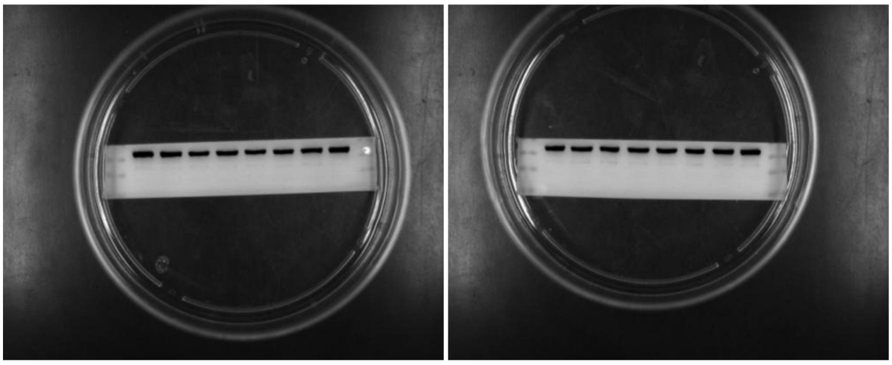

Figure 2B

UCK2

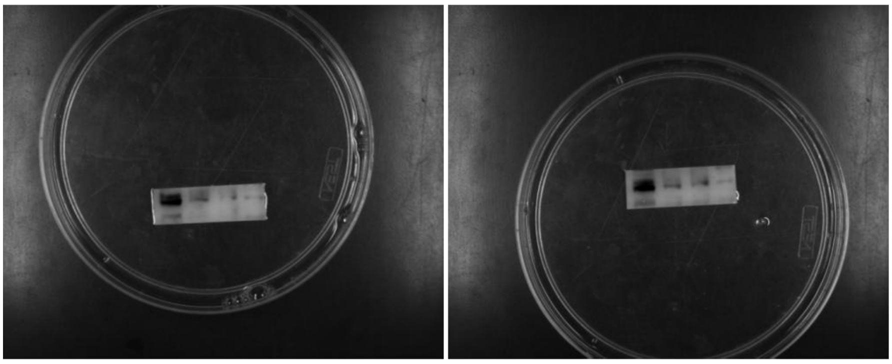

β-actin

Supplemental Material for western blots

Figure 1D

UCK2

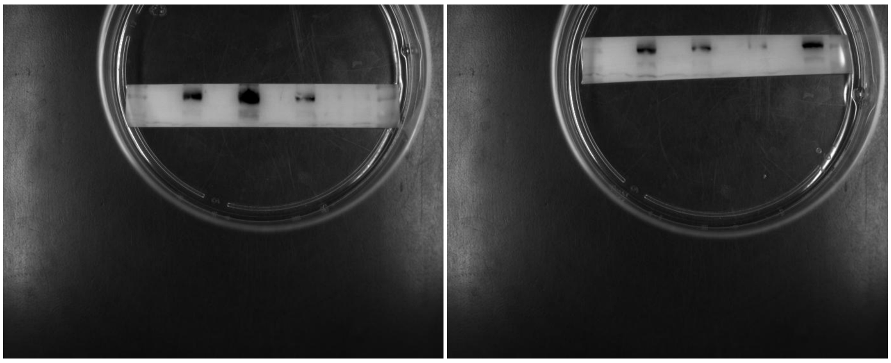

GAPDH

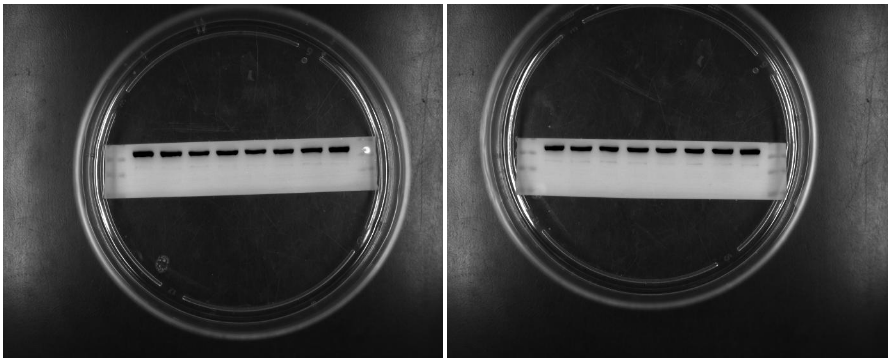

Figure 2B

UCK2

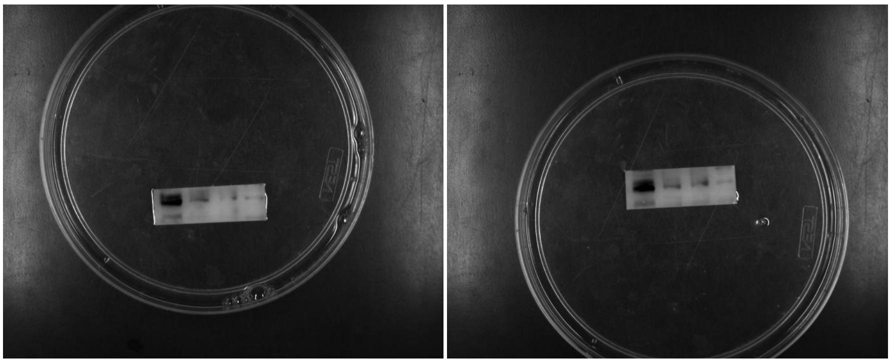

β-actin

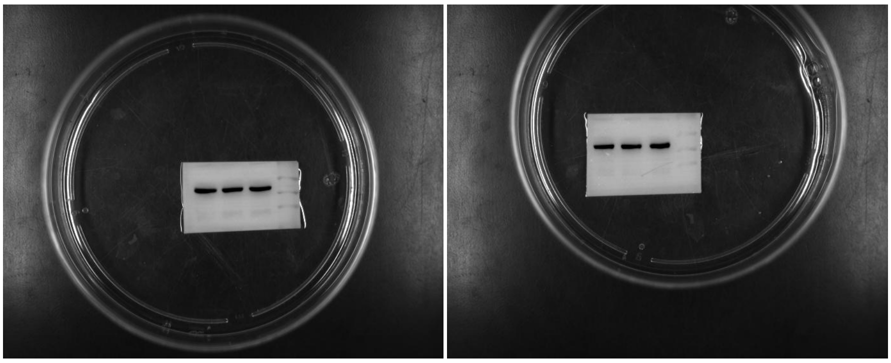

Figure 3B

UCK2

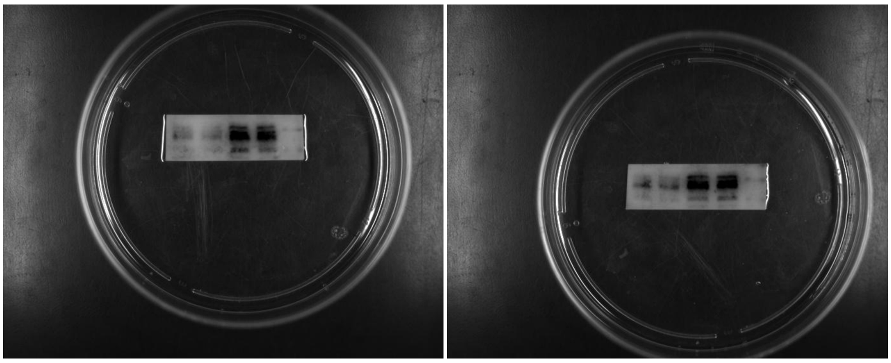

β-actin

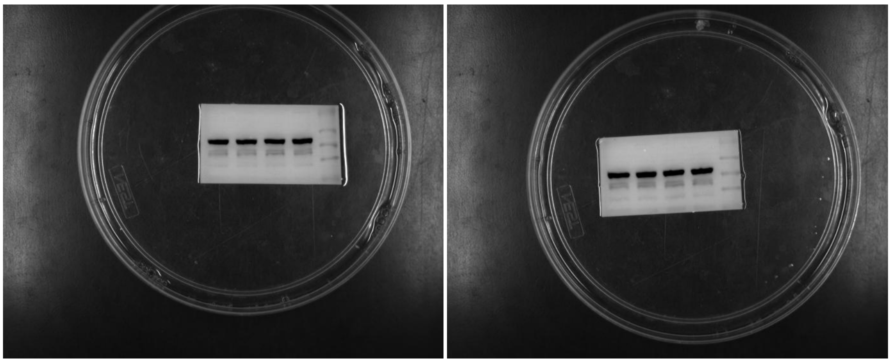

Figure 4D

ShControl shUCK2

PI3K

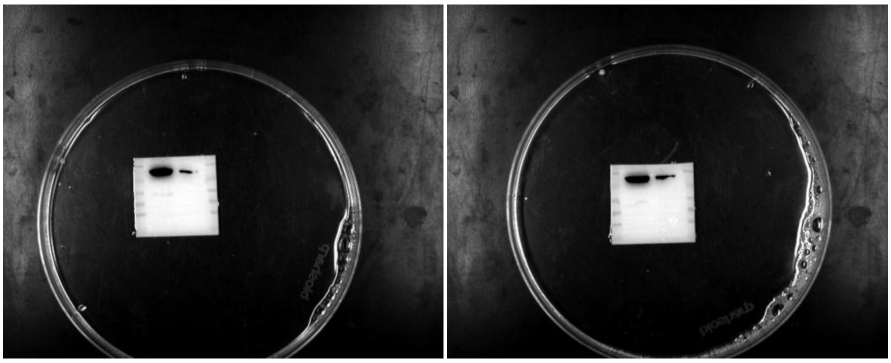

p-AKT

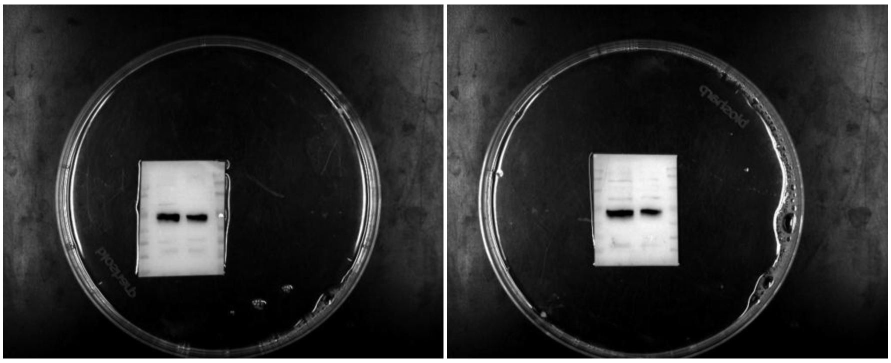

AKT

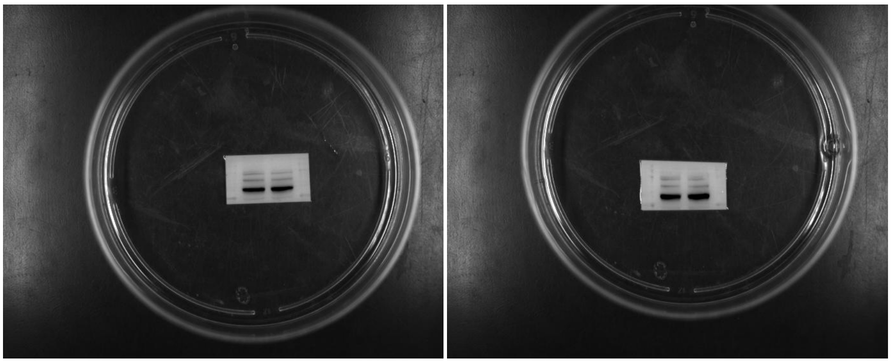

p-mTOR

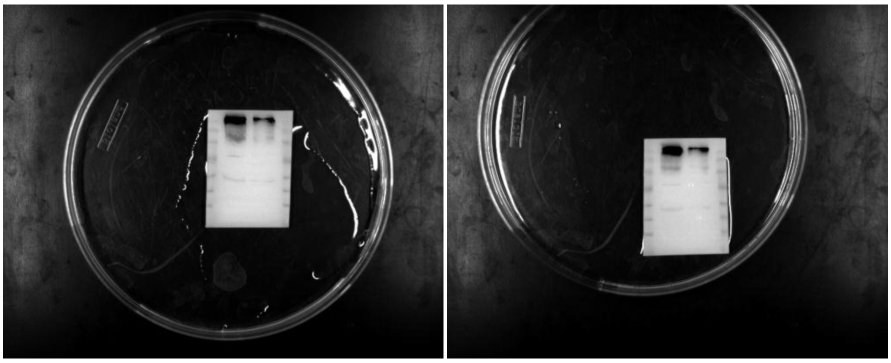

mTOR

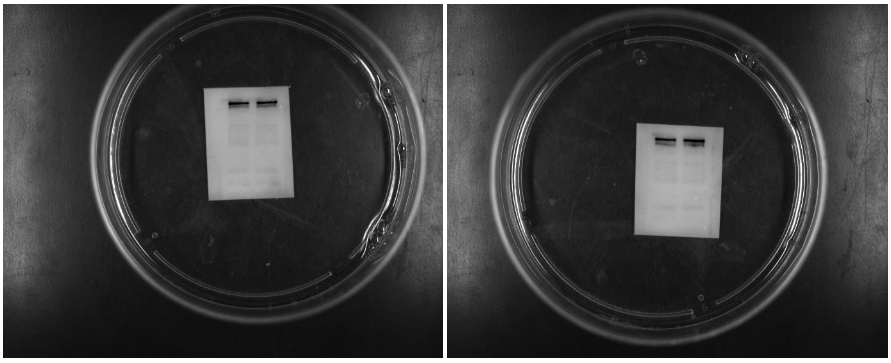

β-actin

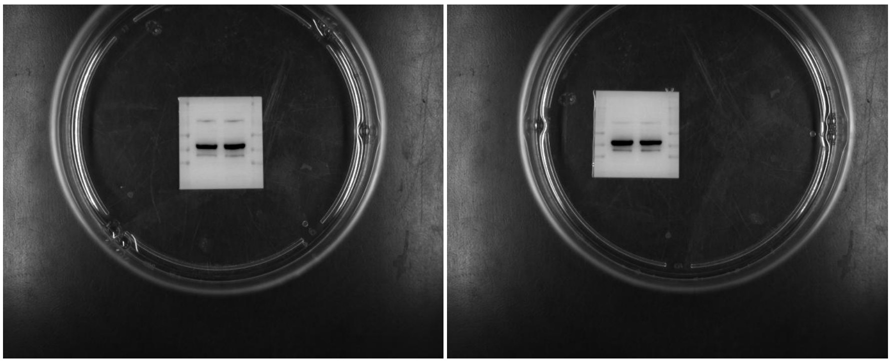

Vector UCK2

PI3K

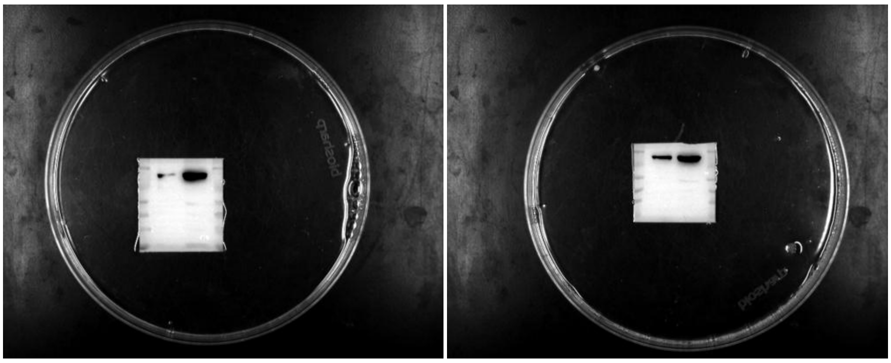

p-AKT

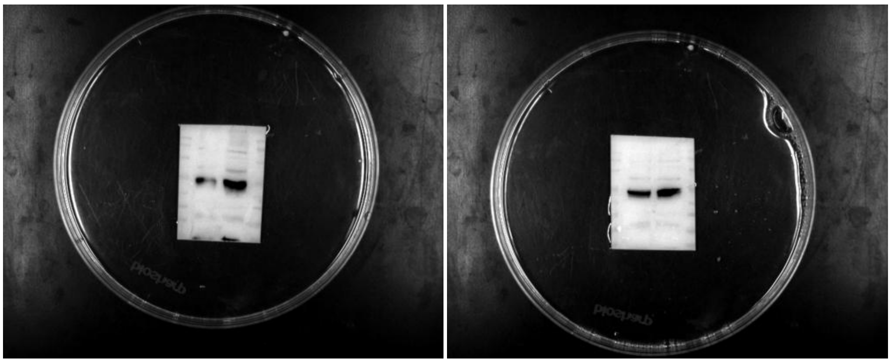

AKT

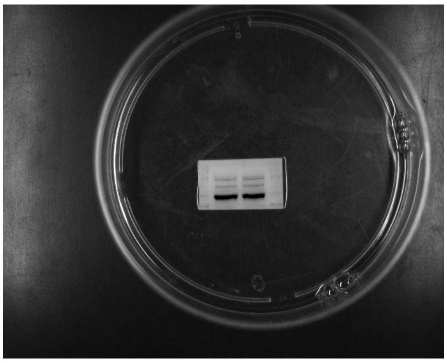 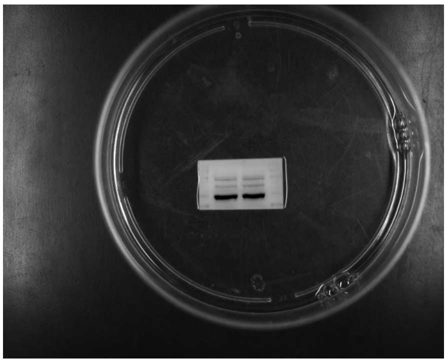

p-mTOR

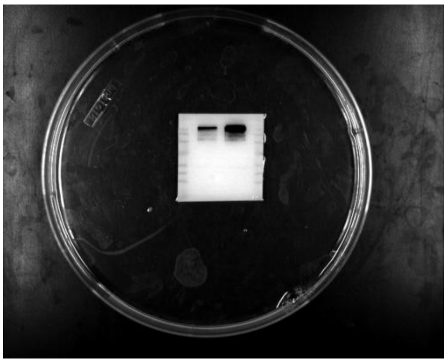 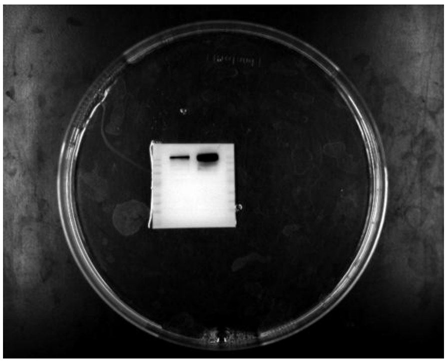

mTOR

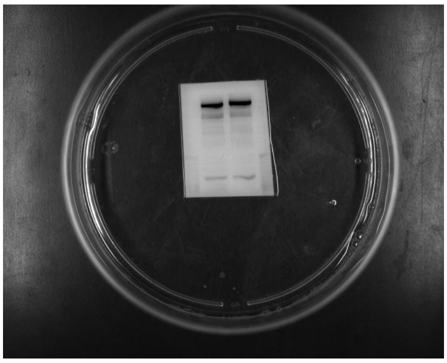 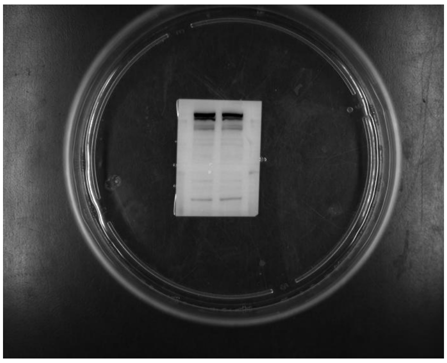

β-actin

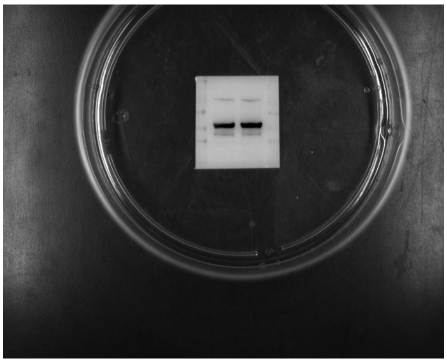 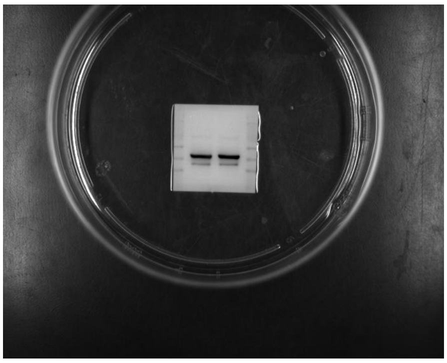

Figure 4 F

ShControl shUCK2

PI3K

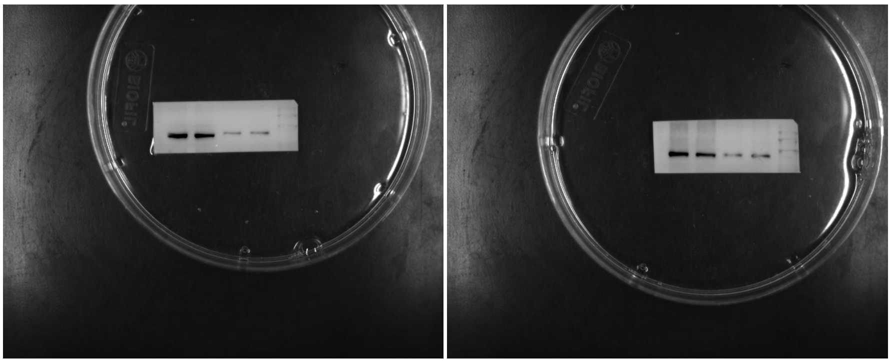

p-AKT

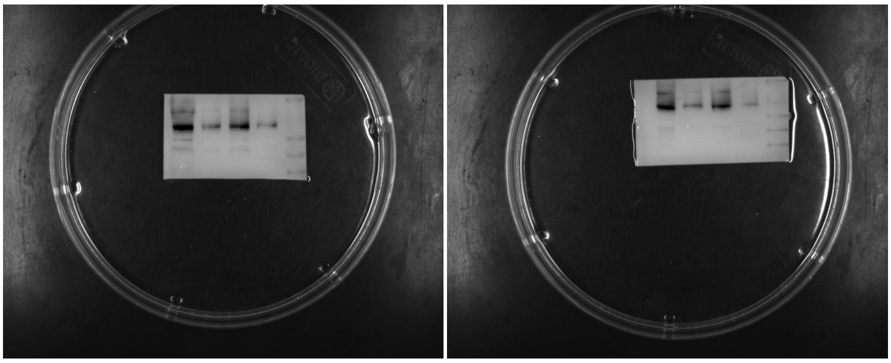

AKT

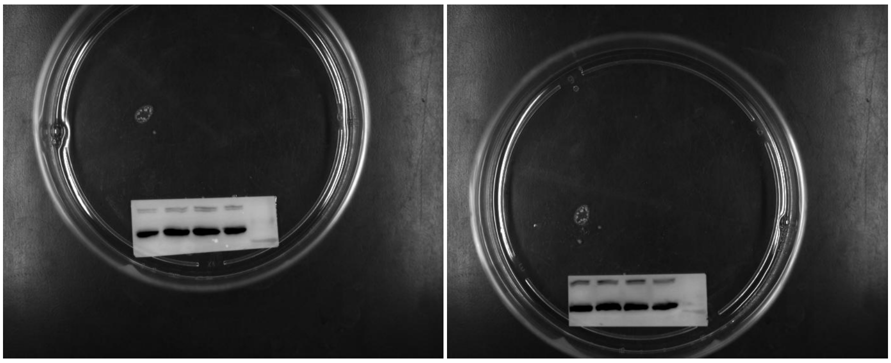

p-mTOR

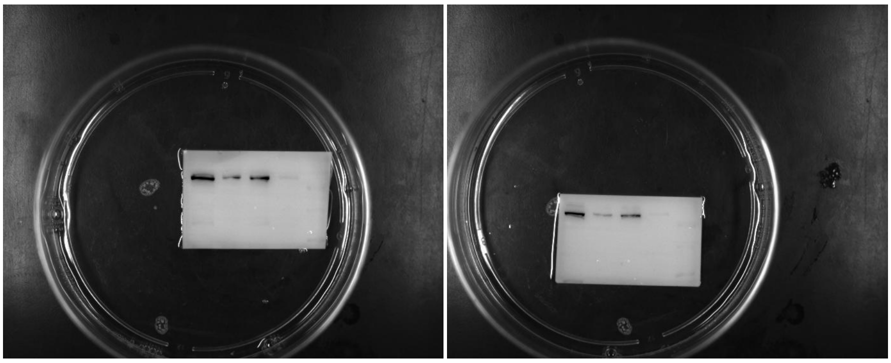

mTOR

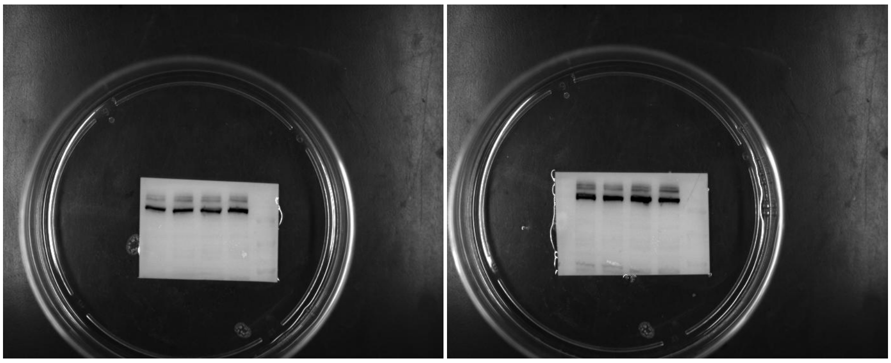

β-actin

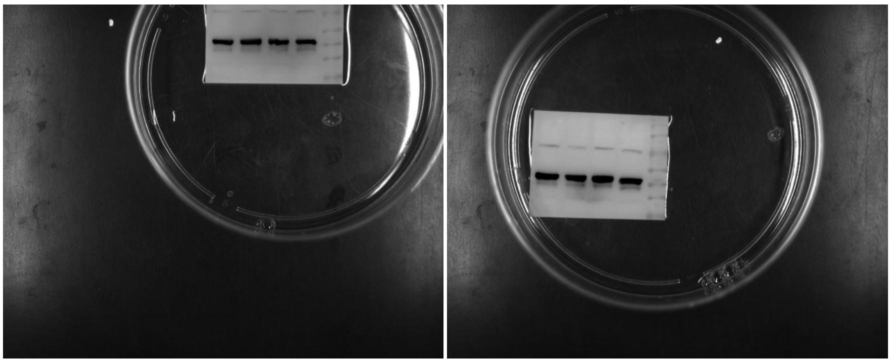

Vector UCK2

PI3K

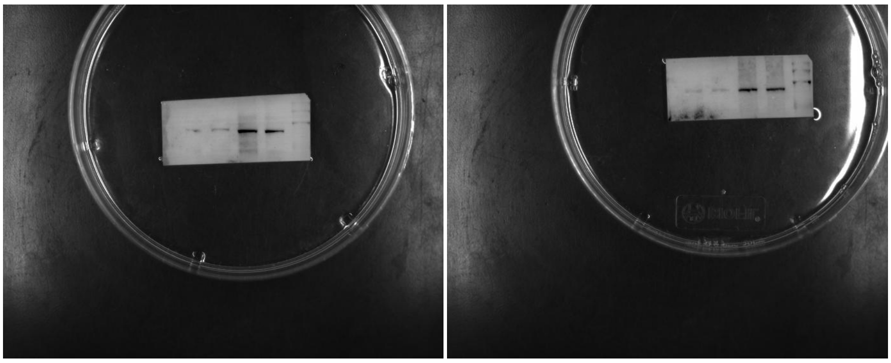

p-AKT

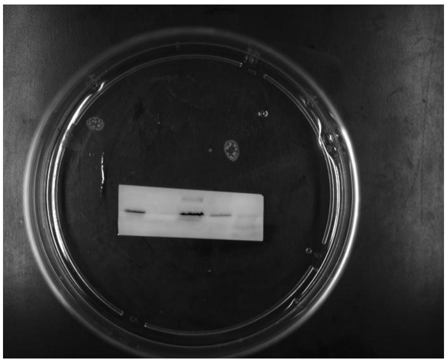
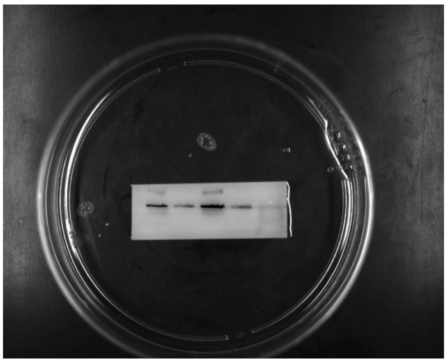

AKT

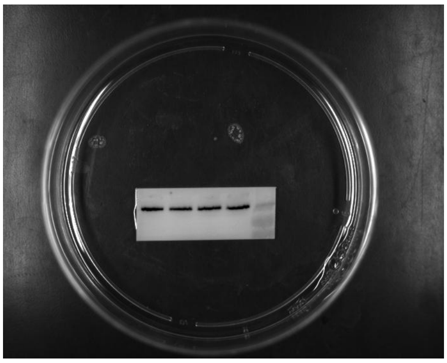
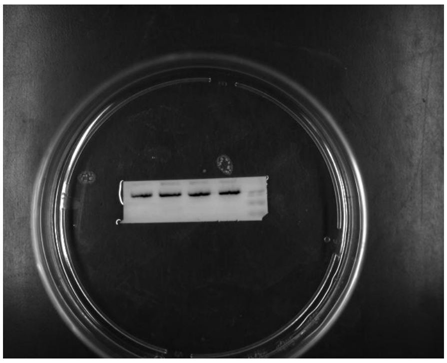

p-mTOR

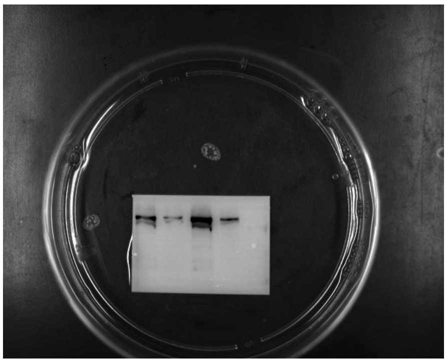
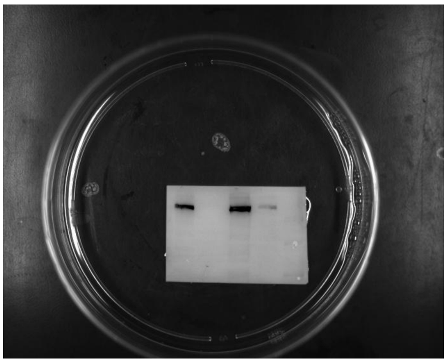

mTOR

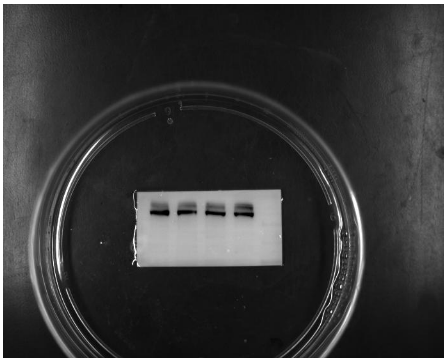
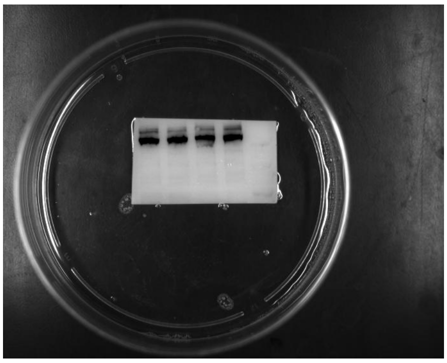

β-actin

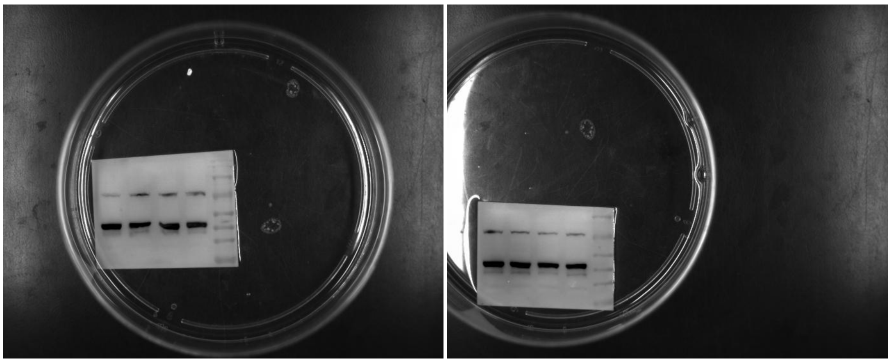

Figure 5A

shControl shUCK2

LC3II/I

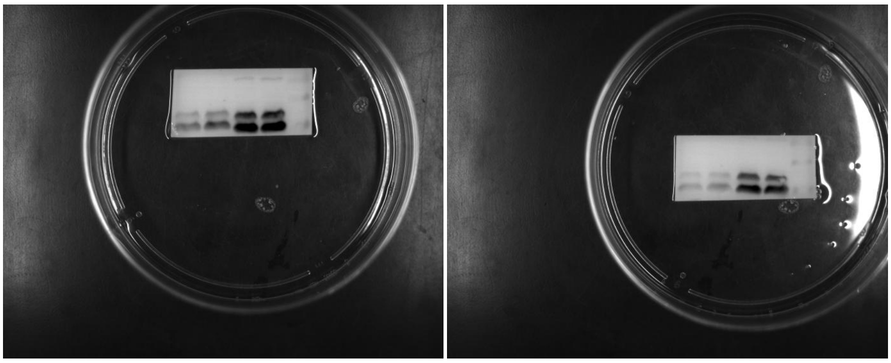

P62

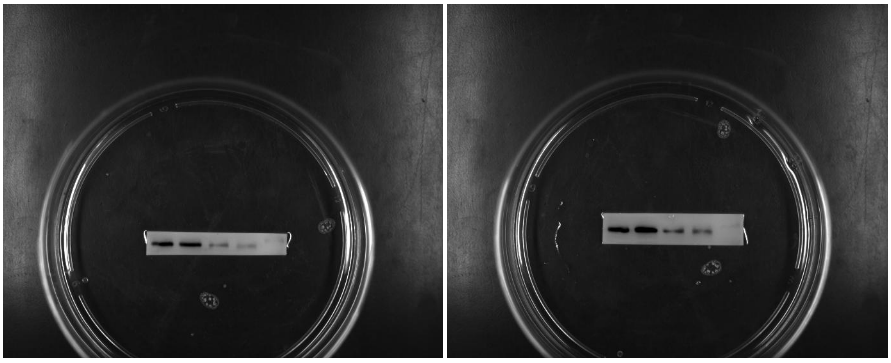

GAPDH

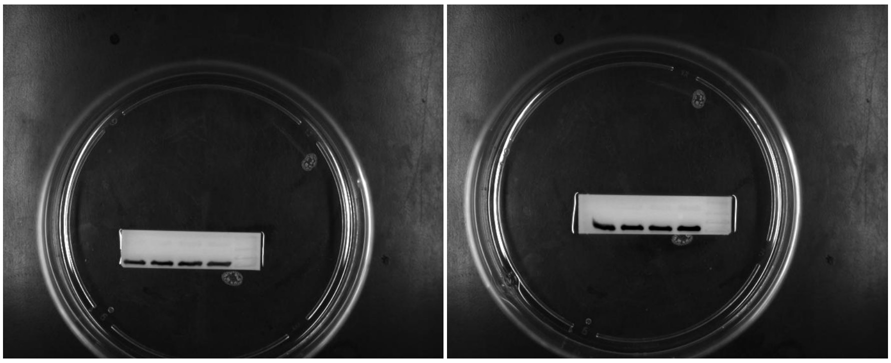

Vector UCK2

LC3II/I

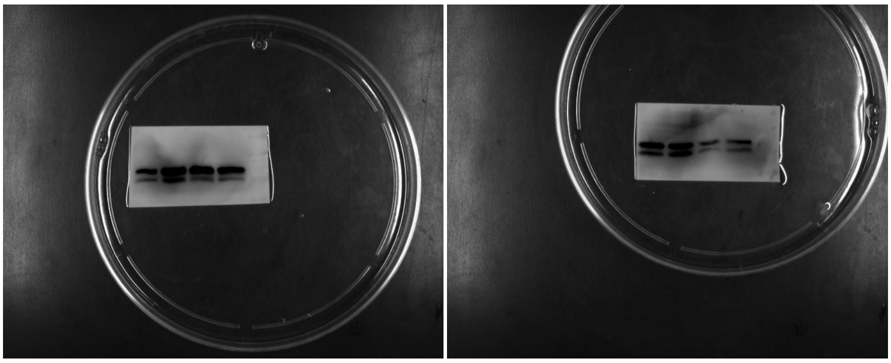

P62

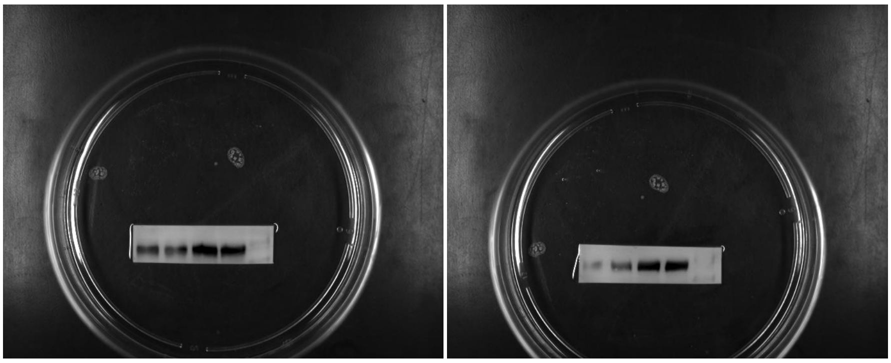

GAPDH

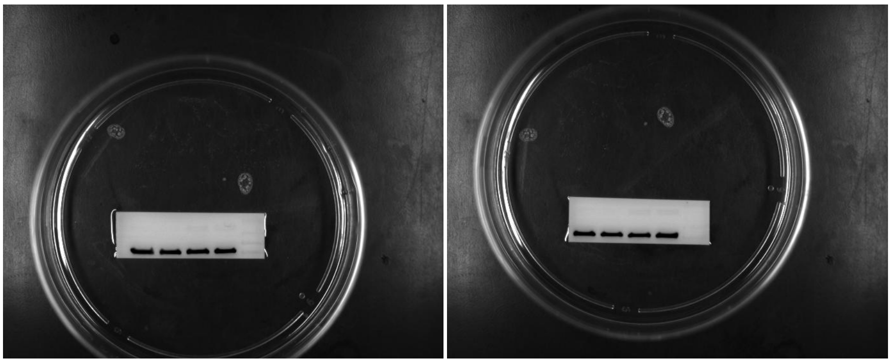

Figure 5E

shControl shUCK2

LC3II/I

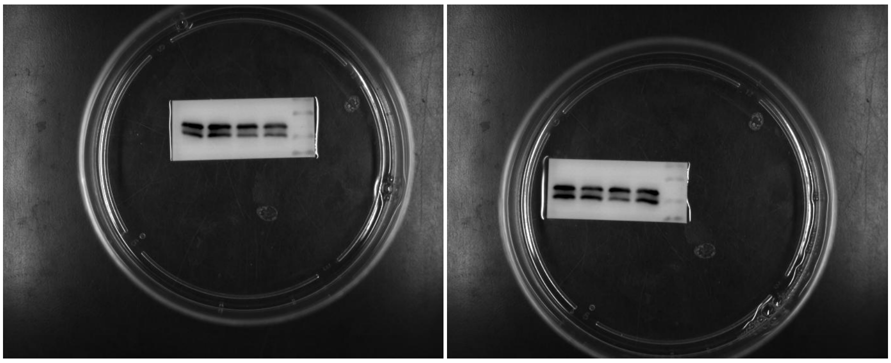

P62

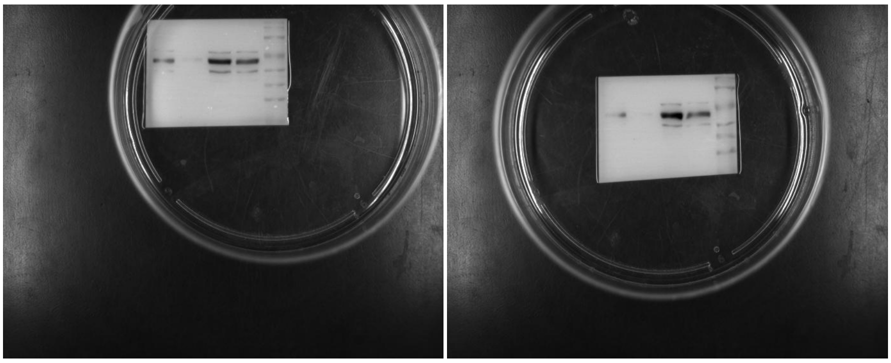

β-actin

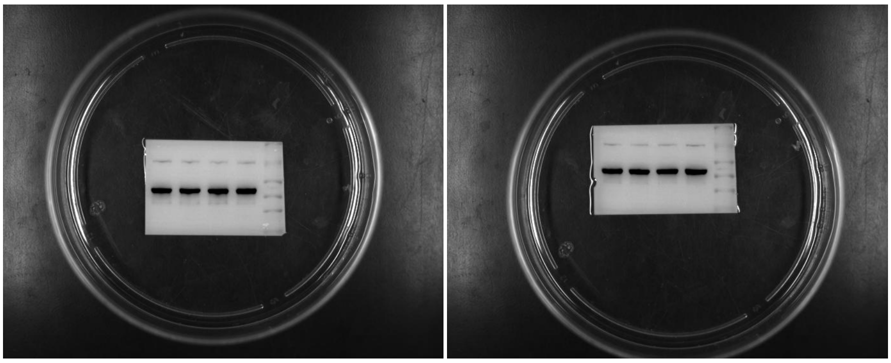

Figure 5F

Vector UCK2

LC3II/I

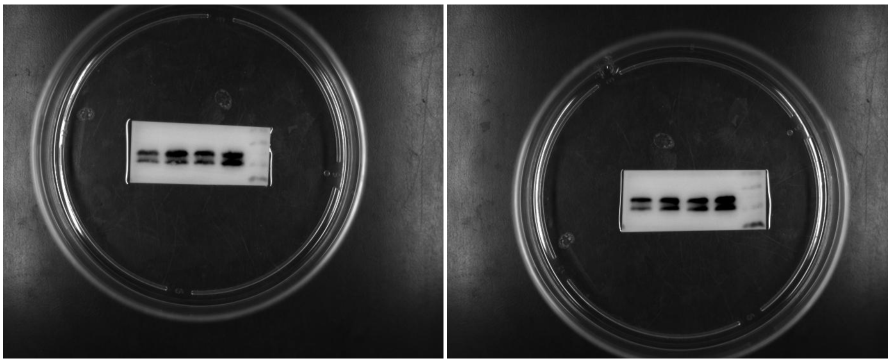

P62

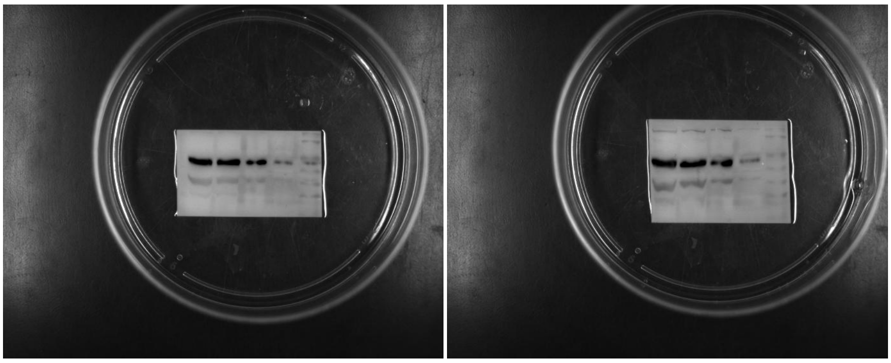

β-actin

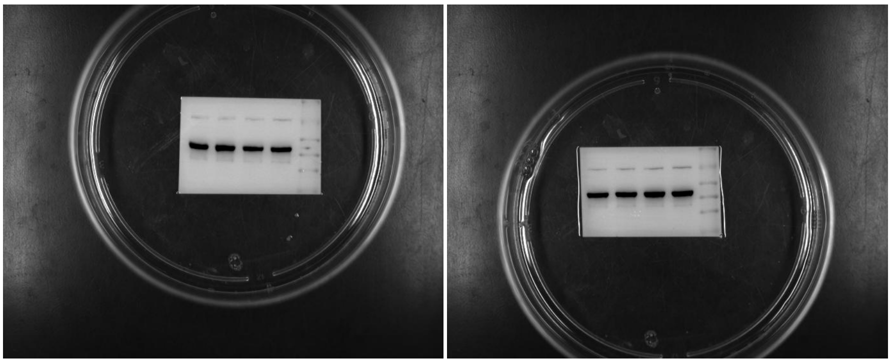

Figure 5G

Vector UCK2

LC3II/I

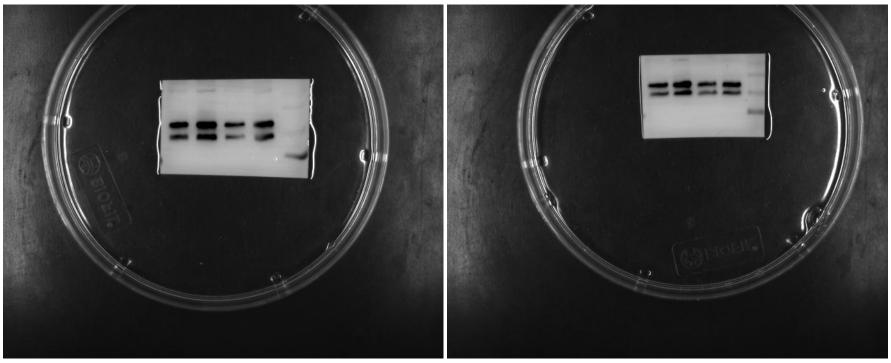

P62

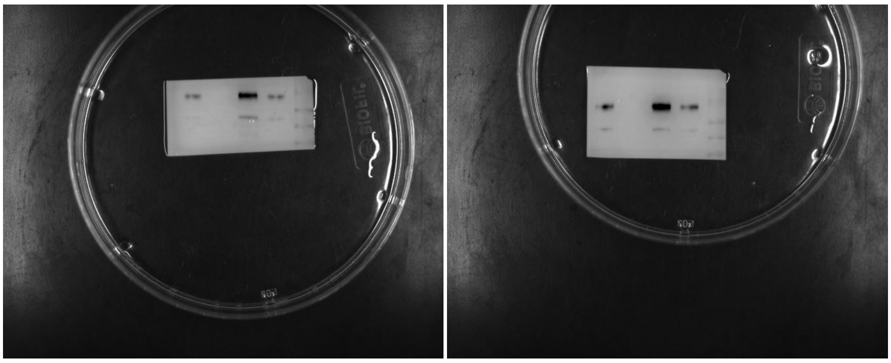

β-actin

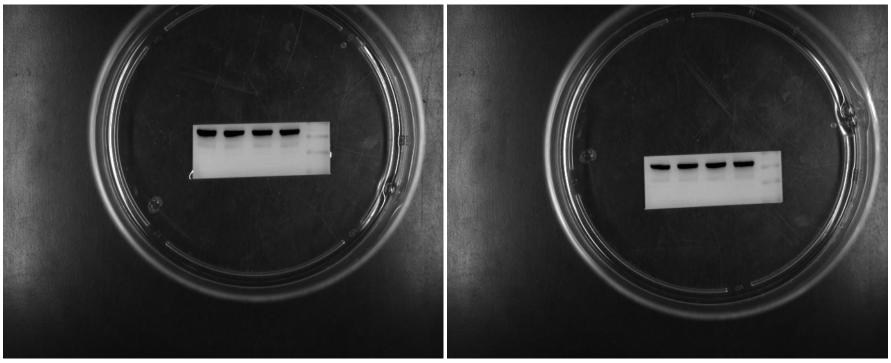

Figure 6B

UCK2

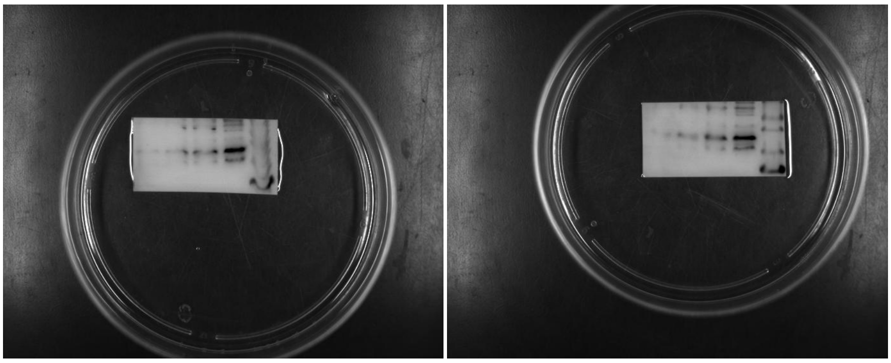

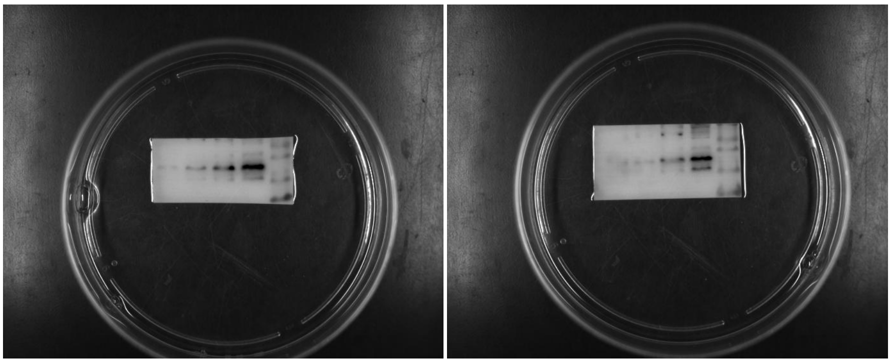

β-actin

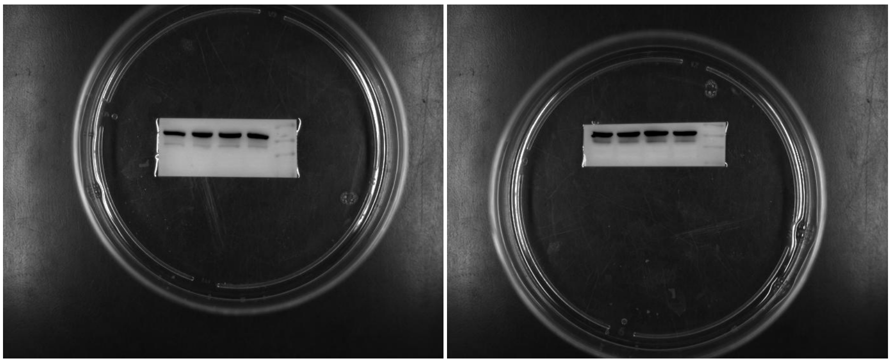

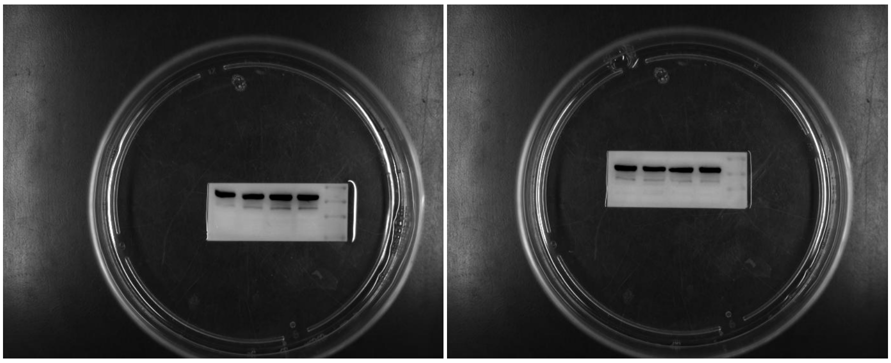

Figure 6C

γ-H2AX

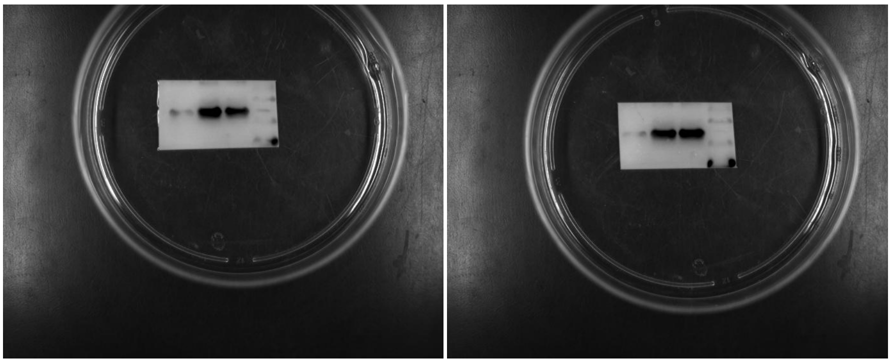

β-actin

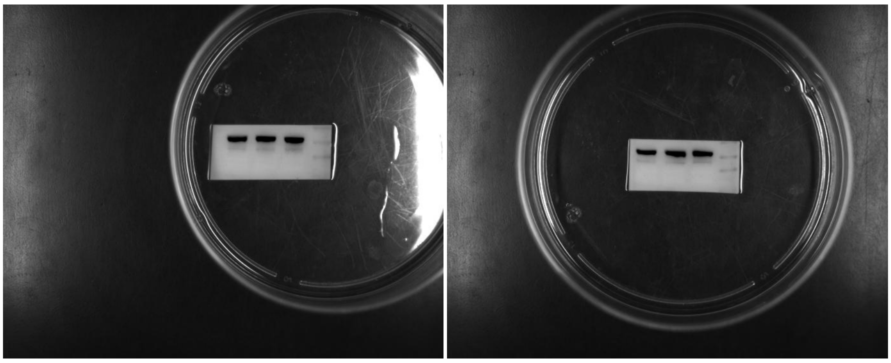

Figure 6F

γ-H2AX

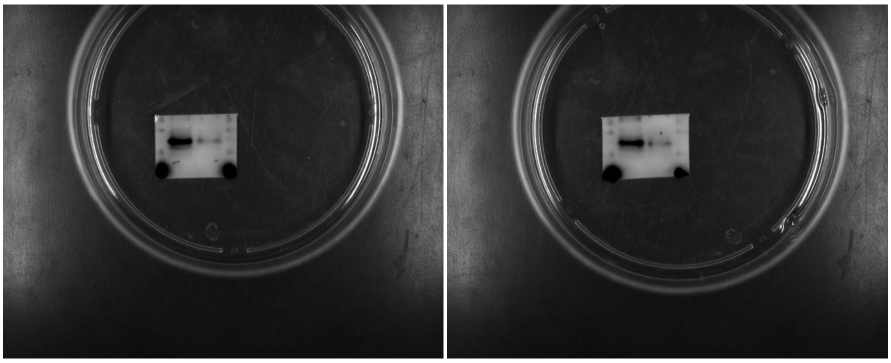

β-actin

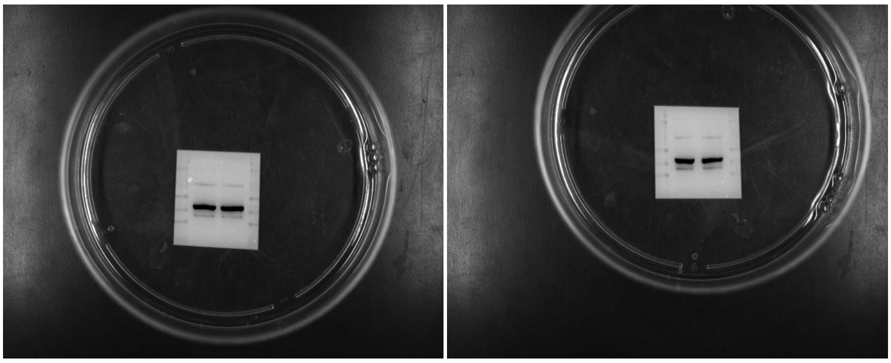

Figure S1A

MK-2206

ShControl shUCK2

PI3K

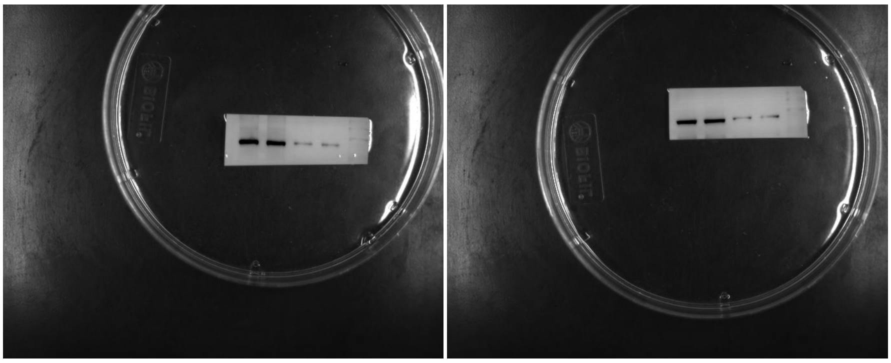

p-AKT

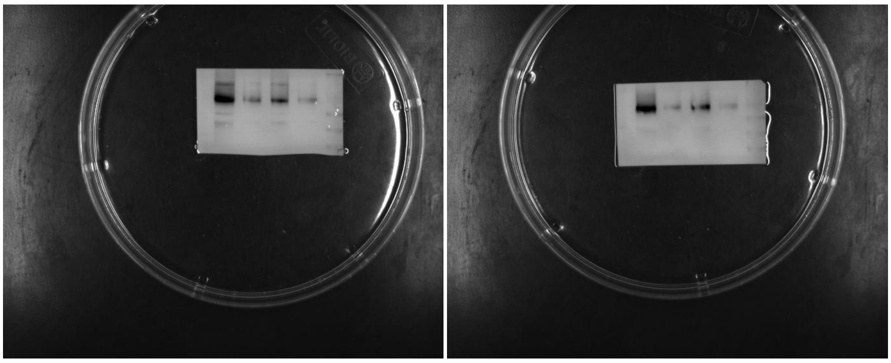

AKT

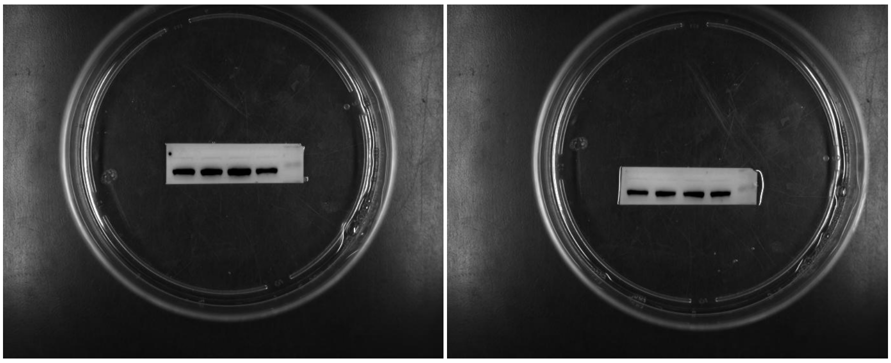

p-MTOR

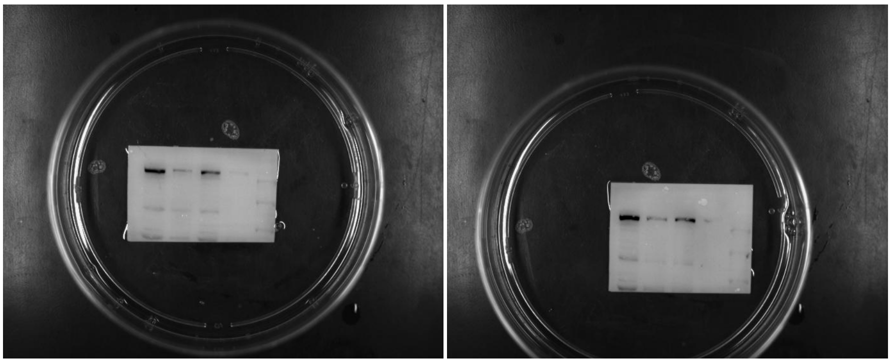

MTOR

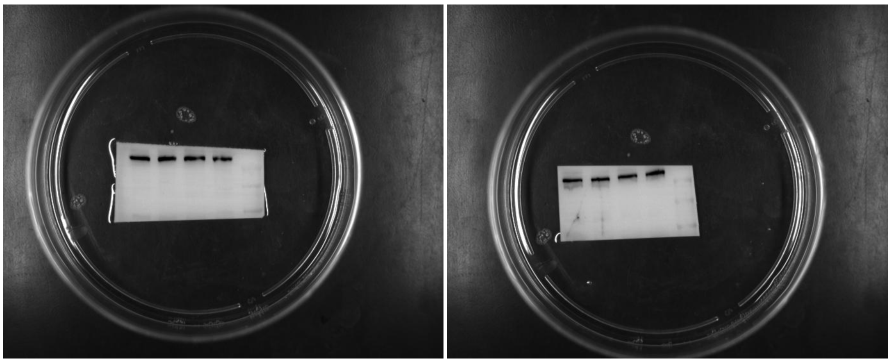

β-actin

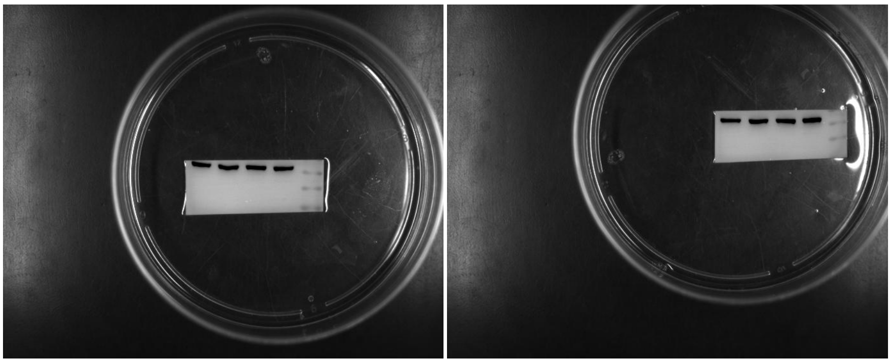

Vector UCK2

PI3K

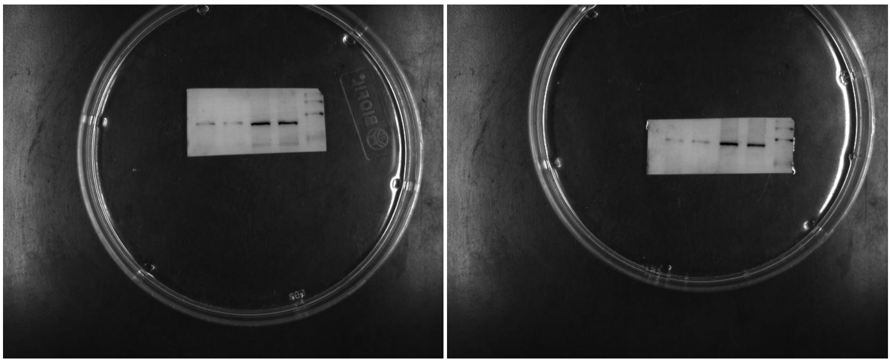

p-AKT

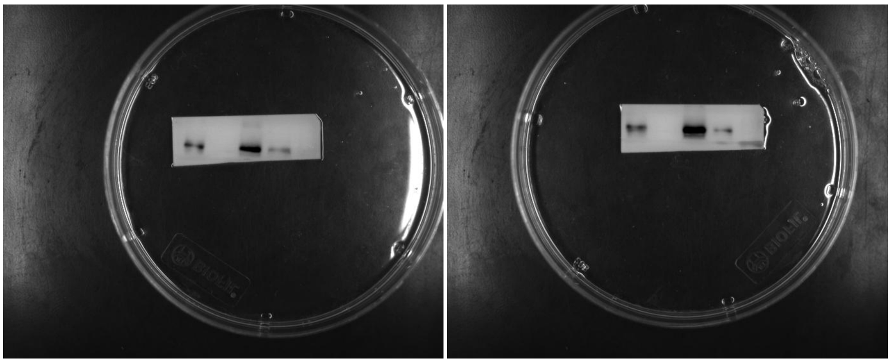

AKT

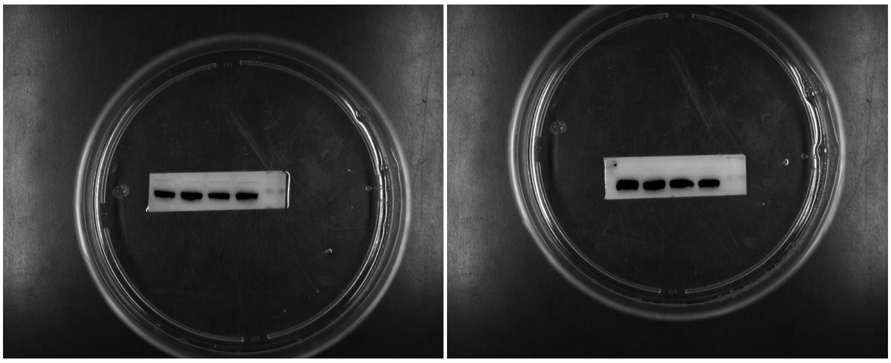

p-MTOR

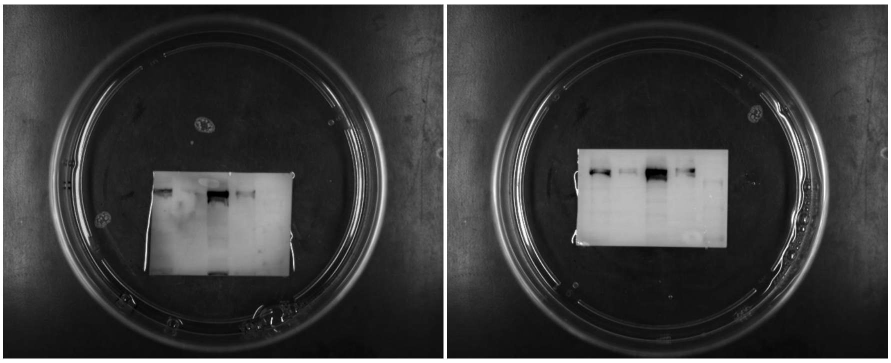

MTOR

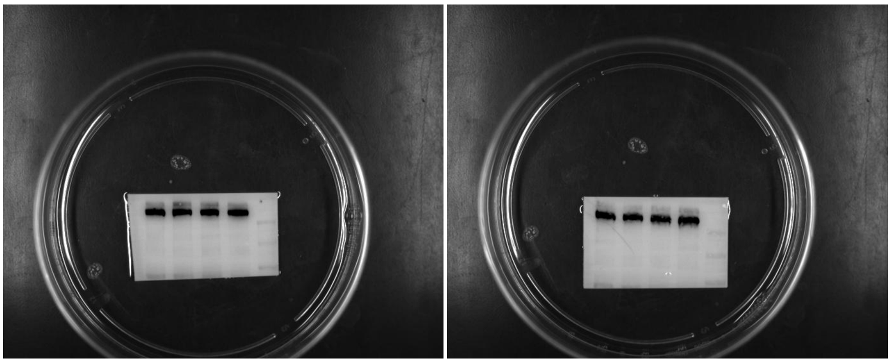

β-actin

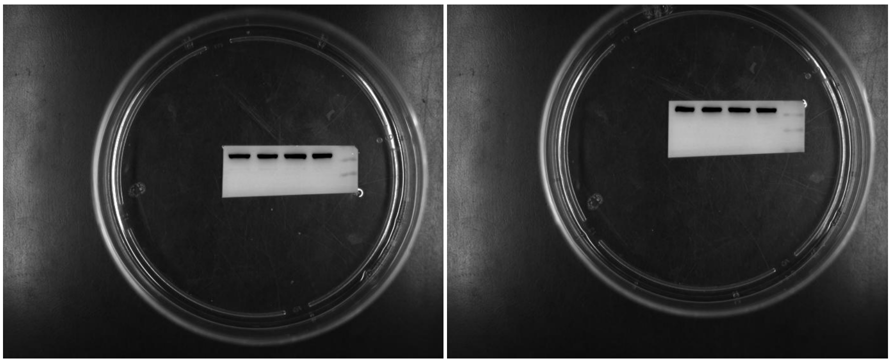

Figure S1B

GDC-0941

ShControl shUCK2

PI3K

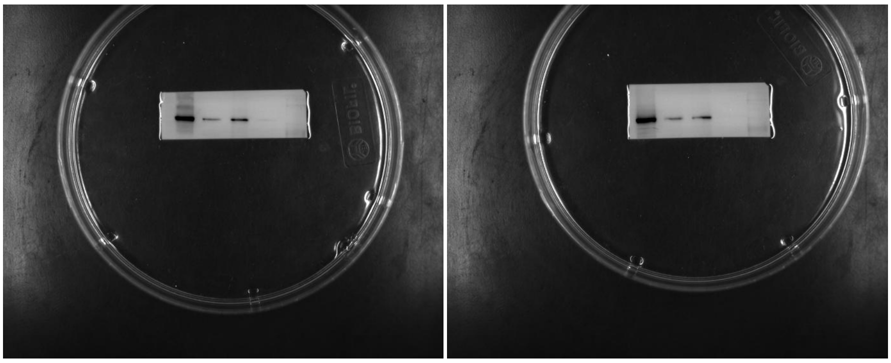

p-AKT

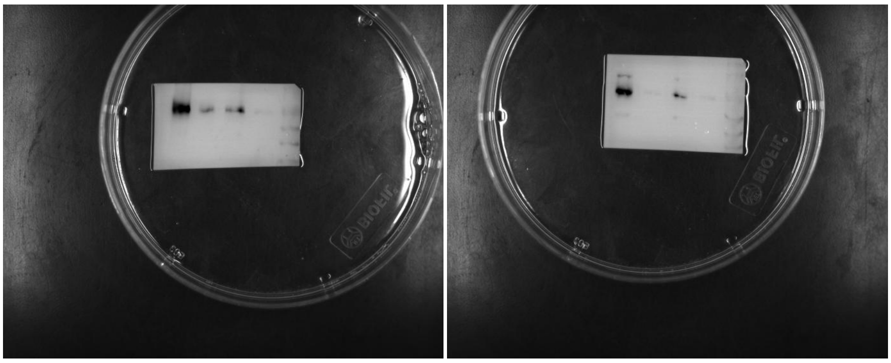

AKT

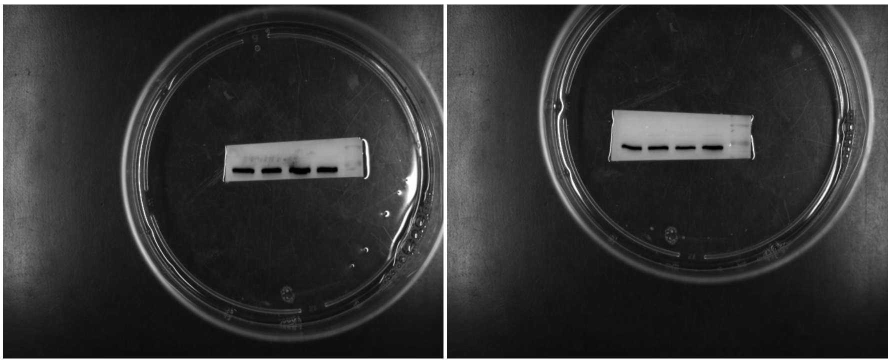

p-MTOR

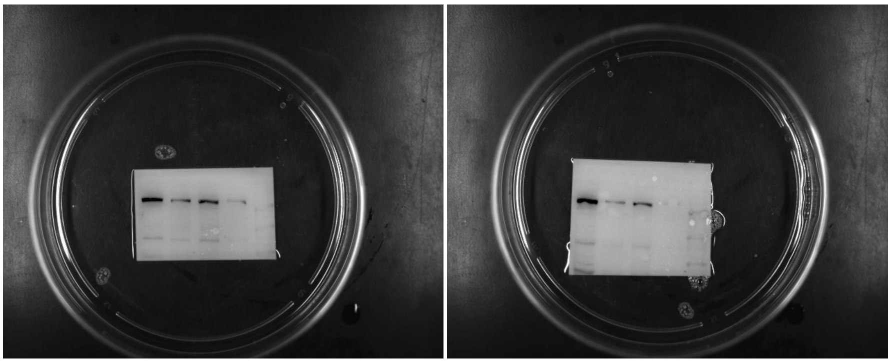

MTOR

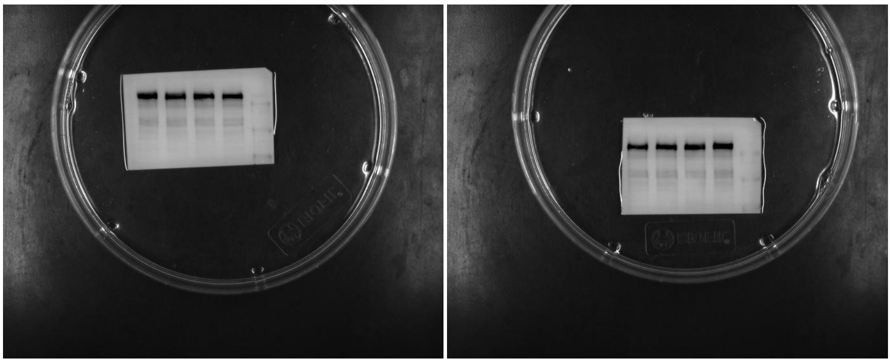

β-actin

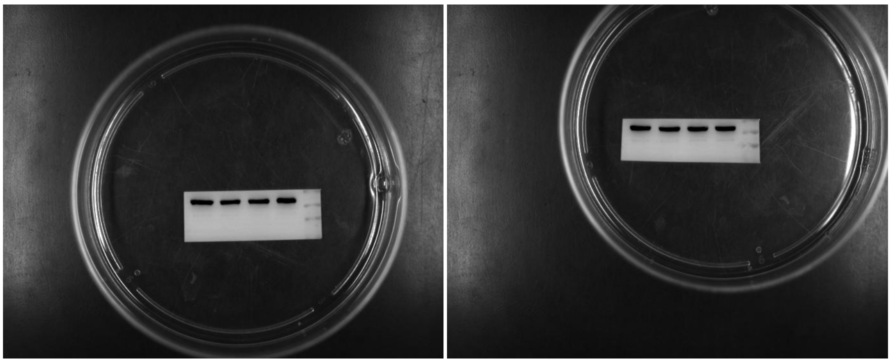

Vector UCK2

PI3K

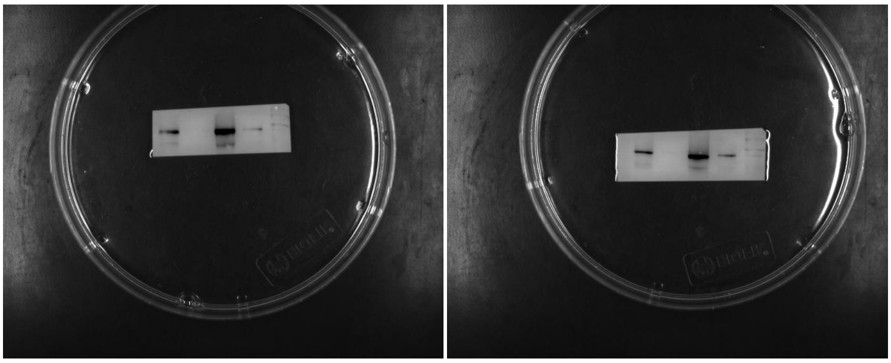

p-AKT

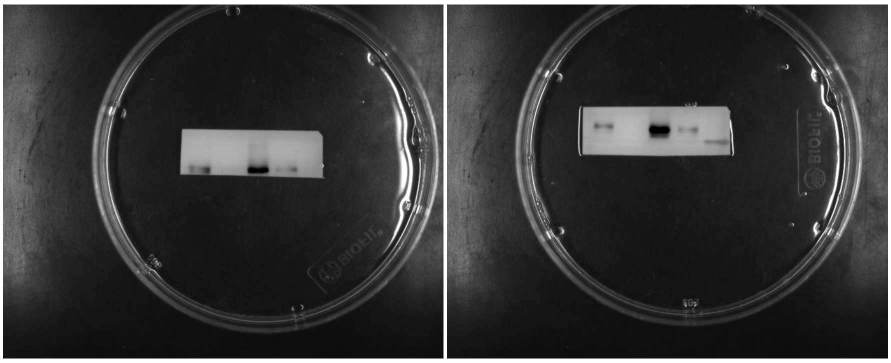

AKT

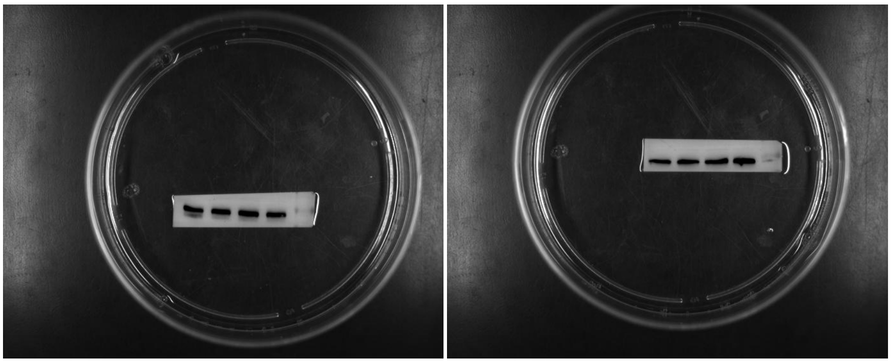

p-MTOR

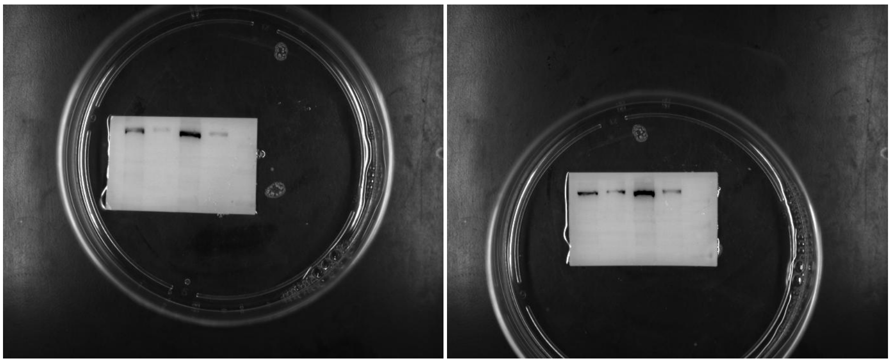

MTOR

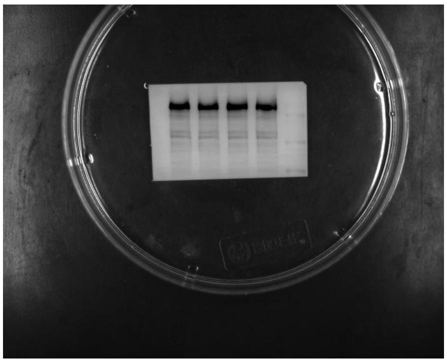

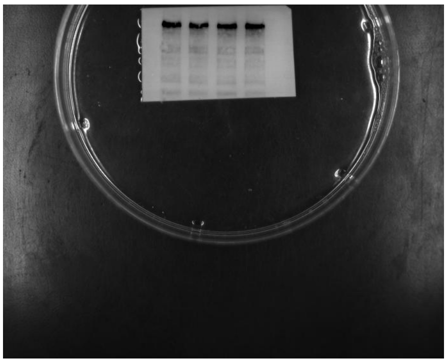

β-actin

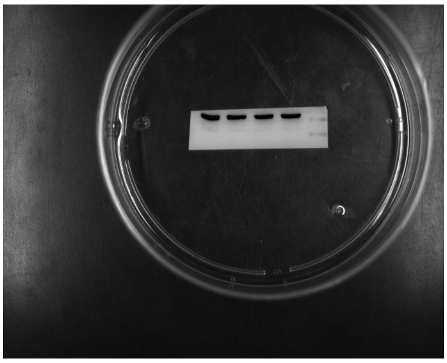

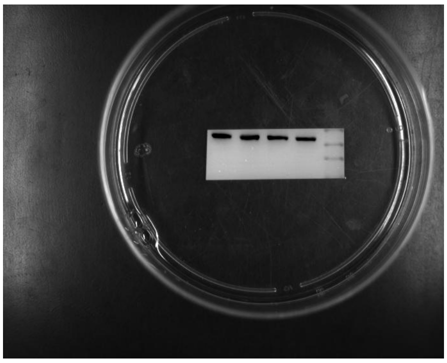

Supplement: Supplementary file 2 — Supplemental Material Revised [file 41420_2024_2140_MOESM2_ESM.pdf]
